# Up‐regulation of *GhTT2‐3A* in cotton fibres during secondary wall thickening results in brown fibres with improved quality

**DOI:** 10.1111/pbi.12910

**Published:** 2018-04-02

**Authors:** Qian Yan, Yi Wang, Qian Li, Zhengsheng Zhang, Hui Ding, Yue Zhang, Housheng Liu, Ming Luo, Dexin Liu, Wu Song, Haifeng Liu, Dan Yao, Xufen Ouyang, Yaohua Li, Xin Li, Yan Pei, Yuehua Xiao

**Affiliations:** ^1^ Biotechnology Research Center Chongqing Key Laboratory of Application and Safety Control of Genetically Modified Crops Southwest University Chongqing China; ^2^ College of Agronomy and Biological Science and Technology Southwest University Chongqing China; ^3^ Institute of Xinjiang Naturally‐Coloured Cotton China Coloured Cotton (Group) Company Urumchi Xinjiang Uygur Autonomous Region China

**Keywords:** *Gossypium*, TT2, proanthocyanidin, brown fibre, transgenic cotton

## Abstract

Brown cotton fibres are the most widely used naturally coloured raw materials for the eco‐friendly textile industry. Previous studies have indicated that brown fibre pigments belong to proanthocyanidins (PAs) or their derivatives, and fibre coloration is negatively associated with cotton productivity and fibre quality. To date, the molecular basis controlling the biosynthesis and accumulation of brown pigments in cotton fibres is largely unknown. In this study, based on expressional and transgenic analyses of cotton homologs of *Arabidopsis* PA regulator TRANSPARENT TESTA 2 (TT2) and fine‐mapping of the cotton dark‐brown fibre gene (*Lc1*), we show that a TT2 homolog, *GhTT2‐3A*, controls PA biosynthesis and brown pigmentation in cotton fibres. We observed that GhTT2‐3A activated GhbHLH130D, a homolog of *Arabidopsis* TT8, which in turn synergistically acted with GhTT2‐3A to activate downstream PA structural genes and PA synthesis and accumulation in cotton fibres. Furthermore, the up‐regulation of *GhTT2‐3A* in fibres at the secondary wall‐thickening stage resulted in brown mature fibres, and fibre quality and lint percentage were comparable to that of the white‐fibre control. The findings of this study reveal the regulatory mechanism controlling brown pigmentation in cotton fibres and demonstrate a promising biotechnological strategy to break the negative linkage between coloration and fibre quality and/or productivity.

## Introduction

Naturally coloured cotton (NCC) undergoes pigmentation in the field by synthesizing and accumulating natural pigments in developing fibres. Compared to white cotton and chemical fibres, NCC bypasses dyeing and bleaching during textile processing, which significantly reduces the release of toxic pollutants and energy and water costs, as well as effectively eliminates harmful chemical residuals in fabrics (Efe *et al*., 2009; Hua *et al*., 2009; Kimmel and Day, 2001). With the increasing demand for green products, environmental protection and human health in modern society, NCCs have attracted interest in terms of their potential use in the textile industry. However, only two types of NCCs (brown and green) are currently commercialized, as green fibres generally fade upon exposure to sunlight (Ma *et al*., 2016). Furthermore, NCC fibres are negatively correlated with fibre quality and productivity (Efe *et al*., 2009; Feng *et al*., 2015; Hua *et al*., 2009; Zhang *et al*., 2009). Consequently, NCCs fail to satisfy the requirements of major consumers and producers and only occupy a small niche in the cotton and textile market. To improve the colour, productivity and/or fibre quality of NCCs and to broaden their applications, it is essential to elucidate the biochemical and molecular bases of pigmentation in NCC fibres and its participation in fibre development.

Brown cotton fibres are the most commonly used NCCs. Extensive efforts have been devoted to characterizing the biochemical properties and the biosynthetic pathway for brown pigments in cotton fibres (Feng *et al*., 2013, 2014; Gong *et al*., 2014; Hinchliffe *et al*., 2016; Hua *et al*., 2007; Li *et al*., 2013; Murthy, 2001; Xiao *et al*., 2007, 2014). Earlier biochemical analyses suggested that flavonoids were involved in the brown coloration of cotton fibres (Hua *et al*., 2009; Murthy, 2001). Transcriptomic and proteomic analyses indicated that a series of structural genes of phenylpropanoid and flavonoid pathways, including those encoding two proanthocyanidin (PA)‐specific enzymes [leucoanthocyanidin reductase (LAR) and anthocyanidin reductase (ANR)], were up‐regulated in brown fibres (Feng *et al*., 2013, 2014; Gong *et al*., 2014; Hinchliffe *et al*., 2016; Li *et al*., 2013; Xiao *et al*., 2007, 2014). Furthermore, the up‐regulation of PA structural genes co‐segregated with brown fibre in a recombinant inbred line (RIL) population (Xiao *et al*., 2007, 2014), suggesting that PA biosynthesis and accumulation were responsible for the brown coloration in cotton fibres. Similarly, dimethylaminocinnamaldehyde (DMACA) and vanillin staining showed that high amounts of PAs accumulated in brown fibres but not in white fibres (Feng *et al*., 2014; Hinchliffe *et al*., 2016; Xiao *et al*., 2007). Biochemical analyses by mass spectrometry (MS) revealed that the main PA monomers in brown cotton fibres contained three hydroxyls on the B ring (gallocatechin or epigallocatechin) (Feng *et al*., 2014; Xiao *et al*., 2014). Feng *et al*. (2014) have demonstrated that PA accumulation in brown fibres starts at an early stage (5 days postanthesis, DPA) and peaks at 30 DPA, whereas in mature brown fibres, PAs are converted to oxidized derivatives (quinones). Because developing brown fibres do not exhibit distinct coloration until maturation, it has been proposed that quinones, instead of their PA precursors, directly contribute to brown pigmentation in cotton fibres (Feng *et al*., 2014). Besides natural coloration, brown NCC fibres also have enhanced flame retardancy (FR) compared to traditional white fibres, rendering them suitable for specific end‐use applications, such as automobile interiors (Hinchliffe *et al*., 2015, 2016; Nam *et al*., 2016; Parmar and Chakraborty, 2001). A recent study has demonstrated that the greater FR in brown NCCs may be the result of increased inorganic salts, such as sodium and potassium, which are possibly sequestered by PAs or PA precursors via metal‐flavonoid complexes (Hinchliffe *et al*., 2016; Nam *et al*., 2016). Therefore, PA biosynthesis plays a key role in conferring brown pigmentation and natural FR to cotton fibres, although PAs may not be the direct source of the brown coloration and/or enhanced FR.

Proanthocyanidins (PAs), also known as condensed tannins, are an important class of flavonoids that are ubiquitously distributed in plants (He *et al*., 2008; Winkel‐Shirley, 2001). Chemically, PAs are oligomers or polymers of flavan‐3‐ol units, which include a large number of compounds differing in monomeric composition, polymerization degree and linkage patterns between units (He *et al*., 2008). Based on genetic and molecular studies in *Arabidopsis*, the regulatory mechanism of PA biosynthesis and accumulation has been elucidated (Appelhagen *et al*., 2014; He *et al*., 2008; Lepiniec *et al*., 2006; Winkel‐Shirley, 2001; Zhao *et al*., 2010). It is proposed that the structural genes encoding PA synthases or transporters are transcriptionally activated by a tripartite complex that is composed of R2R3‐MYB (TT2), bHLH (TT8) and WD‐repeat (TTG1) proteins (Baudry *et al*., 2004, 2006; He *et al*., 2008; Koes *et al*., 2005; Nesi *et al*., 2001). Among these, TT2 is the unique transcriptional regulator of PA biosynthesis, whereas TT8 and TTG1 are involved in a wider scope of processes, including anthocyanin biosynthesis and trichome differentiation (Koes *et al*., 2005). Furthermore, TT2 homologs have been demonstrated to enhance PA biosynthesis in a variety of species, including poplar, grapevine, apple, cacao, persimmon, strawberry and *Lotus japonicus* (Akagi *et al*., 2009; Bogs *et al*., 2007; Gesell *et al*., 2014; Koyama *et al*., 2014; Liu *et al*., 2014, 2015; Mellway *et al*., 2009; Schaart *et al*., 2013; Terrier *et al*., 2009; Wang *et al*., 2017; Yoshida *et al*., 2008), suggesting that the key role of TT2 in PA regulation is highly conserved in higher plants.

Traditional genetic analyses have revealed six loci (*Lc1–Lc6*) controlling brown fibre that are incompletely dominant to white fibre (Kohel, 1985). Using simple sequence repeat (SSR) markers, *Lc1* was mapped to the long arm of chromosome A07 of *Gossypium hirsutum*, and *Lc2* was mapped to the short arm of chromosome A06 (Hinchliffe *et al*., 2016; Li *et al*., 2012; Wang *et al*., 2014; Zhang *et al*., 2005, 2009). Recently, *Lc1* has been linked to a 1.4‐Mb inversion upstream of a TT2 homologous gene (*GhTT2_A07*) on chromosome A07 (Hinchliffe *et al*., 2016). Although expression analyses have indicated a correlation between high‐level expression of *GhTT2_A07* with PA accumulation, pigmentation and enhanced FR in brown cotton fibres, genetic evidence elucidating the biological functions of this gene is limited. Furthermore, the *Lc1* completely linked fragment contained over 100 annotated genes in *G. raimondii* (*Gorai.001G009900–00G020500*), including two other TT2 homologs (*Gorai.001G020400 and Gorai.001G020500*) that were tandemly linked to the *GhTT2_A07* paralog (*Gorai.001G020600*). To identify the determinant regulator for brown fibre, this study performed functional analysis of cotton TT2 homologs, fine‐mapping of the *Lc1* gene, and generated transgenic brown fibre cottons by up‐regulating *GhTT2‐3A* (*GhTT2_A07*), particularly in fibres at the secondary wall‐thickening stage.

## Results

### Identification, expression and functional analysis of TT2 homologs in cotton

Previous studies have indicated that PAs or PA derivatives play essential roles in coloration in brown NCC fibres (Feng *et al*., 2014; Hinchliffe *et al*., 2016; Xiao *et al*., 2007, 2014). Considering that brown fibre is genetically characterized as a qualitative trait that is controlled by a single gene (*Lc1*) in upland cotton T586 (Kohel, 1985; Zhang *et al*., 2009), we envisage that *Lc1* encodes a PA regulator that activates the PA pathway in brown cotton fibres (Xiao *et al*., 2007). Recently, Hinchliffe *et al*. (2016) have reported that a cotton TT2 homolog (*GhTT2_A07*) is linked to brown fibre coloration and high‐level expression of *GhTT2_A07* results in up‐regulation of the PA pathway. In an attempt to characterize the gene responsible for the brown coloration in cotton fibres, we conducted a comprehensive identification and functional analysis of TT2 homologs in cotton.

As shown in Figure S1, a total of 47 *G. raimondii* R2R3‐MYB proteins were annotated as TT2 homologs in the Phytozome collections (https://phytozome.jgi.doe.gov) (Goodstein *et al*., 2012). Phylogenetic reconstruction indicated that five proteins were clustered with TT2 and were thus regarded as putative TT2 homologs (Figures S1 and S2), hereby designated as *GrTT2‐1–GrTT2‐5* (Table 1). Their homologous genes were further identified in the assembled *G. arboreum* and *G. hirsutum* genomes (Table 1). For each *G. raimondii* gene, a single homologous sequence was identified in *G. arboreum* and *G. hirsutum* At1 and Dt1 subgenomes, respectively. All these identified sequences were regarded as TT2 homologs and named according to the reference genes plus species abbreviations and subgenome (A or D) in the tetraploid cotton (Table 1). All these TT2 homologs in *G. raimondii*,* G. arboreum* and *G. hirsutum* were then amplified from their respective genomic DNAs. Sequence analyses indicated that the sequences and gene structures of the homologous genes were highly conserved in different subgenomes of upland cotton and the extant diploid progenitors (Figure S3).

**Table 1:** TT2 homologous genes identified in assembled cotton genomes

References	D5	A2	Dt1	At1
*GoTT2‐1*	*GrTT2‐1Gorai.001G020400* Chr01:1923027*–*1924079	*GaTT2‐1Cotton_A_05642* Ca1:2638844*–26*39991	*GhTT2‐1DGohir.D07G019700* D07: 2130059*–*2131117	*GhTT2‐1AGohir.A07G019900* A07: 2229796*–*2230862
*GoTT2‐2*	*GrTT2‐2Gorai.001G020500* Chr01:1941820*–*1942904	*GaTT2‐2* Ca1:2667878*–*2668980	*GhTT2‐2DGohir.D07G019800* D07: 2143037*–*2144078	*GhTT2‐2AGohir.A07G020000* A07: 2262733*–*2263774
*GoTT2‐3*	*GrTT2‐3Gorai.001G020600* Chr01:1968735*–*1969655	*GaTT2‐3Cotton_A_05641* Ca1:2686427*–*2687402	*GhTT2‐3DGohir.D07G019900* D07: 2167905*–*2168921	*GhTT2‐3AGohir.A07G020200* A07: 2296049*–*2297025
*GoTT2‐4*	*GrTT2‐4Gorai.001G015200* Chr01:1427526*–*1429468	*GaTT2‐4Cotton_A_05698* Ca1:2068904*–*2070858	*GhTT2‐4DGohir.D07G014400* D07: 1603363*–*1605336	*GhTT2‐4AGohir.A07G014700* A07: 1685671*–*1687623
*GoTT2‐5*	*GrTT2‐5Gorai.010G087200* Chr10:13355170*–*13356235	*GaTT2‐5Cotton_A_25175* Ca3:76125284*–*76126349	*GhTT2‐5DGohir.D06G074700* D06: 14592783*–*14593848	*GhTT2‐5AGohir.A06G075700* A06: 19978299*–*19979364

Gene names are indicated with annotated protein‐coding genes and corresponding locations (in bp) annotated in the genome sequencing projects for allotetraploid *G. hirsutum* (Dt1 and At1, https://phytozome.jgi.doe.gov/pz/portal.html#!info?alias=Org_Ghirsutum_er) and its extant diploid progenitors *G. raimondii* (D5) (Paterson *et al*., 2012) and *G. arboreum* (A2) (Li *et al*., 2014). *GaTT2*‐2 is not annotated in the original genome sequencing project.

**Figure 1:**
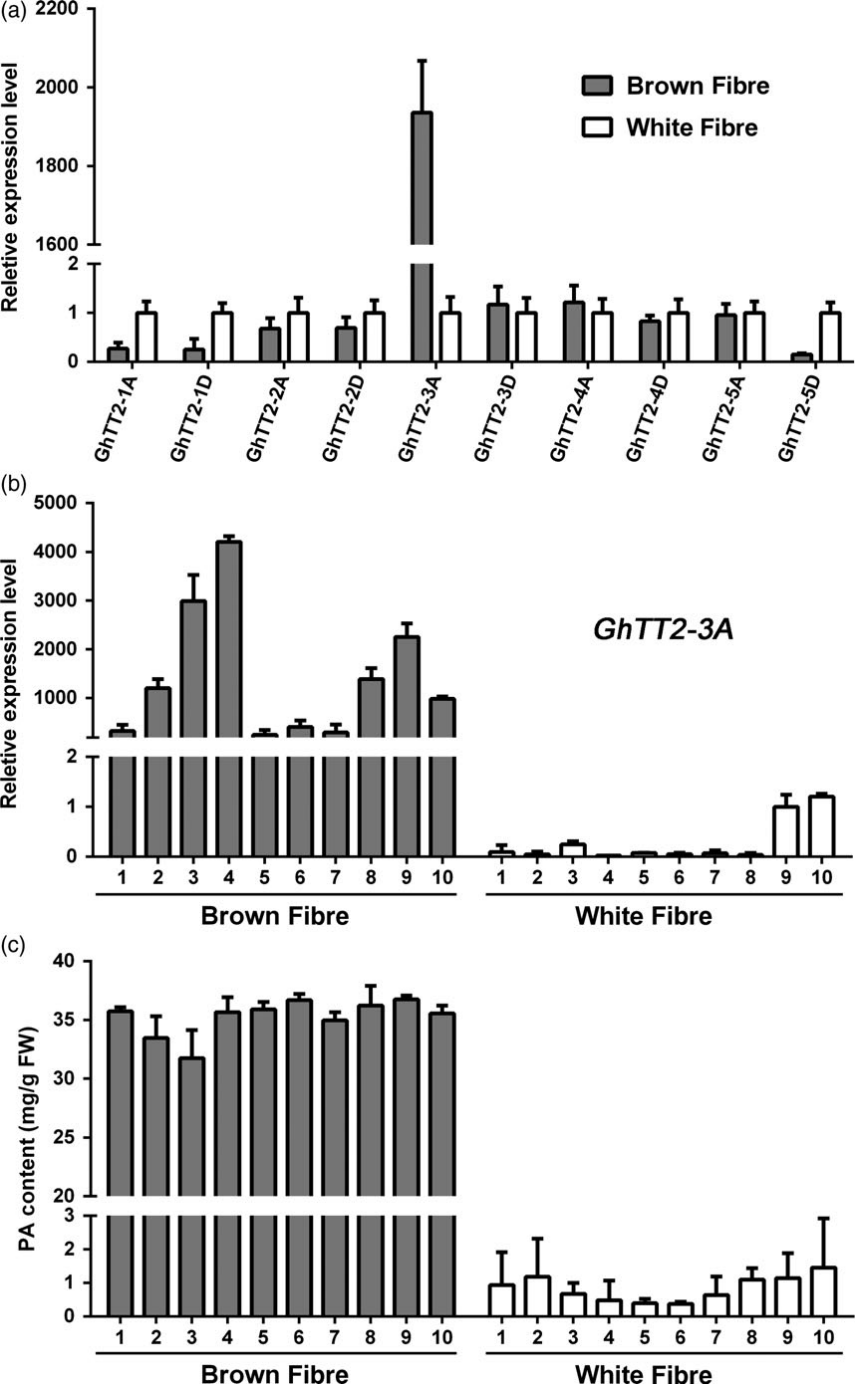
Expression of TT2 homologous genes and PA accumulation in brown and white fibres of RILs. Fibres of 20 DPA of each 10 brown‐ and white‐fibre RILs derived from T586 × Yumian No. 1 were harvested for RNA and PA extraction. (a) qRT–PCR analysis of five pairs of TT2 homologous genes using two bulks generated by equally mixing RNAs from each 10 brown‐ and white‐fibre RILs. The white‐fibre bulk was used as control. (b and c) qRT–PCR analysis of *GhTT2‐3A* expression and PA contents in brown and white fibres from various RILs.

To determine whether cotton TT2 homologs are involved in regulating PA biosynthesis and brown pigmentation in fibres, their transcript and PA levels in developing brown and white fibres are compared. Figure 1 shows that only *GhTT2‐3A* (*Gohir.A07G020200*), which was recently reported as *GhTT2_A07* (Hinchliffe *et al*., 2016), is highly expressed in brown fibres, and the high‐level expression of *GhTT2‐3A* co‐segregated with PA accumulation and brown fibre coloration in the RIL population (Figure 1b,c). Moreover, the high‐level expression of *GhTT2‐3A* coincided with PA accumulation in developing brown fibres, whereas none of the nine other *G. hirsutum* TT2 homologs exhibited significant increased expression in brown fibres compared to white fibres (Figures S4 and S5).

**Figure 2:**
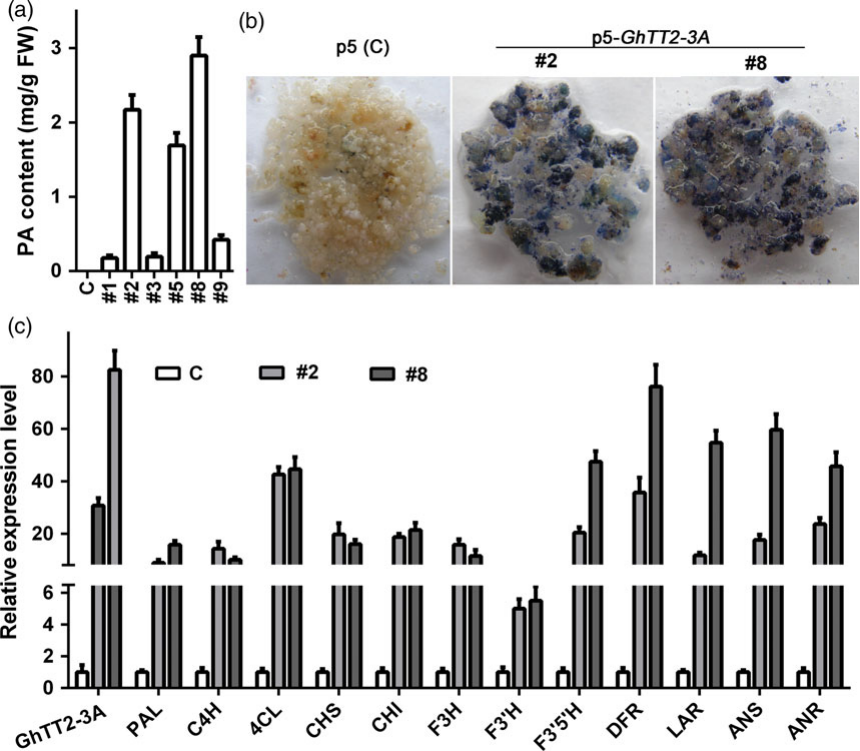
*GhTT2‐3A* promotes PA biosynthesis and accumulation in transgenic cotton calli. Control (c) is the empty vector (p5)‐transformed calli. (a) PA contents in the control and *GhTT2‐3A*‐transformed calli. (b) PA detection using DMACA staining. (c) qRT–PCR analysis of the expression of *GhTT2‐3A* and PA structural genes. Both homologous PA structural genes from At and Dt subgenomes are amplified as indicated in Figure S8. 4CL, 4‐coumarate:CoA ligase; ANR, anthocyanidin reductase; ANS, anthocyanidin synthase; C4H, cinnamate 4‐hydroxylase; CHI, chalcone isomerase; CHS, chalcone synthase; DFR, dihydroflavonol 4‐reductase; F3H, flavone 3‐hydroxylase; F3′H, flavonoid 3′‐hydroxylase; F3′5′H, flavonoid 3′5′‐hydroxylase; LAR, leucoanthocyanidin reductase; PAL, phenylalanine ammonia lyase.

To elucidate the biological functions of cotton TT2 homologs, we first transformed CaMV35S‐driven *GhTT2‐3A* into white‐fibre cotton Jimian No. 14 (J14). No transgenic plant overexpressing *GhTT2‐3A* was obtained, but PAs were up‐regulated in transgenic calli (Figure 2a,b). *GhTT2‐3A* and downstream PA structural genes were significantly up‐regulated in those PA‐accumulating calli (Figure 2c), indicating that GhTT2‐3A activates the entire PA pathway by activating PA structural genes in cotton calli. Furthermore, overexpressing four other *G. hirsutum* TT2 homologous genes (*GhTT2‐1D*,* GhTT2‐2D*,* GhTT2‐4D* and *GhTT2‐5D*) also induced PA accumulation in transgenic calli (Figure S6). Considering that the sequence similarities between homologous genes from different (sub) genomes are higher compared to those between paralogs (Table 1 and Figure S3), these results demonstrate that the TT2 homologs identified in this study are probably all active transcription factors that promote PA biosynthesis and accumulation in cotton.

### Fine‐mapping of *Lc1* in upland cotton

**Figure 3:**
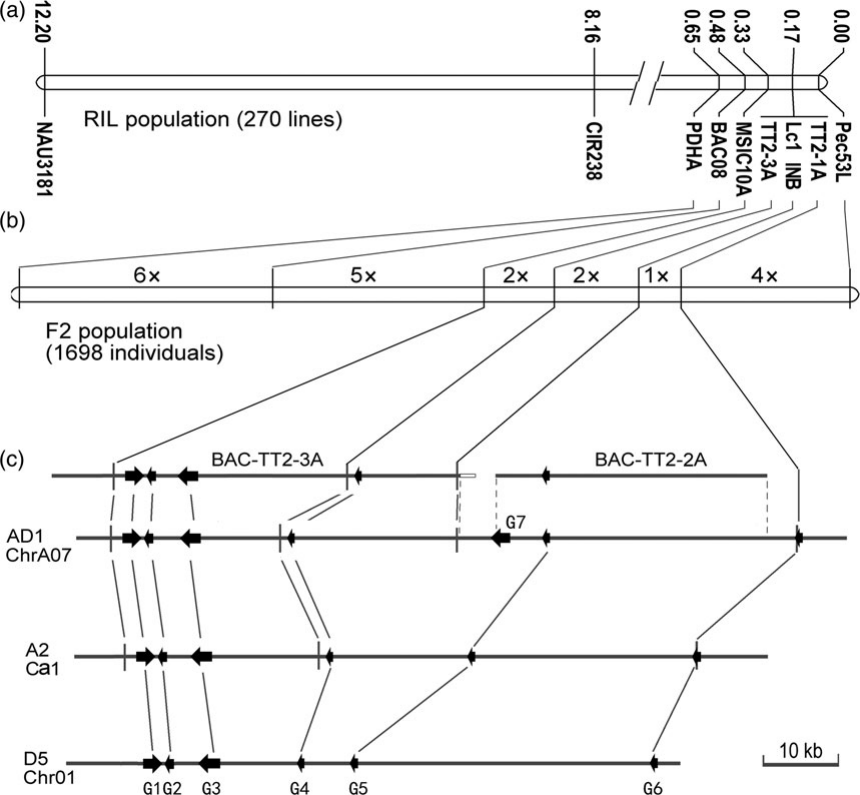
Fine‐mapping of the *Lc1* gene. (a and b) the genetic map of *Lc1* region constructed using RIL and enlarged F2 population, respectively. Markers in A are indicated along with the genetic distance calculated according to recombinant frequency. The map in B is drawn to scale to the genetic distance, with the number of recombination events indicated in the intervals between markers. (c) Contig analysis of *Lc1* region in *G. hirsutum* (AD1) and collinearity with *G. arboreum* (A2) and *G. raimondii* (D5). Two BACs and the corresponding region from *G. hirsutum* genome sequence (JGI v1.1) are aligned. The BACs (BAC‐TT2‐2‐3A and BAC‐TT2‐2A) are screened from the BAC libraries of T586 and Yumian No. 1 using gene‐specific primers (Table S5). The borders of overlapped sequences are indicated by dashed lines. Identical or homologous loci are represented by solid lines. Arrows show annotated protein‐coding genes (Table S2). G1–G6 represent the annotated genes *Gorai.001G020900–001G020400* in *G. raimondii*, respectively. G2 in AD1 and A2, and G5 in A2, which are not annotated in the original genome sequencing projects, are validated according to sequence similarity to *G. raimondii* genes. G7 may be a recently amplified gene, for only one copy was detected in *G. arboreum* (*Cotton_A_32686*) and *G. raimondii* (*Gorai.009G304100*), whereas a total of 16 copies, including the collinear homologs (*Gohir.A05G287800* and *Gohir.D05G289000*), are identified in the assembled *G. hirsutum* genome (Table S2). The open bar at the end of BAC‐TT2‐3A indicates the inversed fragment that is homologous to *Gorai.001G009800* in *G. raimondii*.

Along with the transcriptomic and biochemical analyses of brown‐fibre (*Lc1*) cottons (Xiao *et al*., 2007, 2014), the *Lc1* gene was mapped to chromosome A07 and linked to SSR markers NAU3181 and CIR238 using a RIL population of upland cottons T586 × Yumian No. 1 (Zhang *et al*., 2009). Here, we employed fine‐mapping to clarify the relationship between *Lc1* and TT2 homologs, particularly *GhTT2‐3A* (*GhTT2_A07*), which is highly expressed in brown cotton fibres. To this end, seven markers in the *GhTT2‐3A* region were explored on the basis of comparative cloning between T586 and Yumian No. 1 (Table S1). Using the T586 × Yumian No. 1 RIL population, *Lc1* was mapped between markers Pec53L and MSIC10A and co‐segregated with three markers, namely TT2‐1A, INB and TT2‐3A (Figure 3a). These loci were further separated on the linkage map derived from an enlarged F2 population comprising 1698 individuals. Consequently, the *Lc1* gene co‐segregated with INB and was mapped to a region between TT2‐1A and TT2‐3A (Figure 3b). In the assembled *G. hirsutum* genome, this interval of approximately 67 kb contained three protein‐coding genes (Figure 3c; Table S2), including a ribosomal protein L9 gene and two TT2 homologs (*GhTT2‐2A* and *GhTT2‐3A*). Because only *GhTT2‐3A* is significantly expressed in brown fibres and its up‐regulation promotes PA biosynthesis and accumulation in cotton (Figures 1, 2, S4 and S5), it is possible that *GhTT2‐3A* is the determinant gene of cotton brown fibres, as previously reported by Hinchliffe *et al*. (2016).

### Specific up‐regulation of *GhTT2‐3A* in cotton fibres

**Figure 4:**
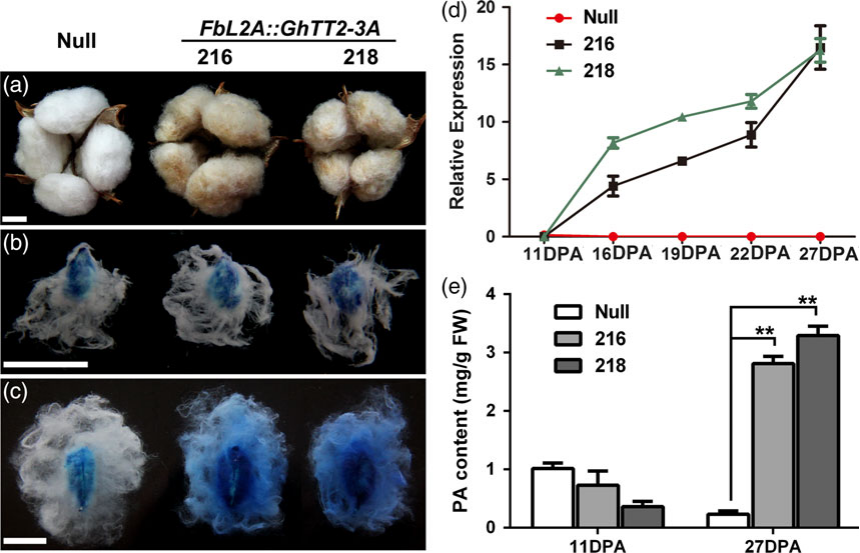
Up‐regulation of *GhTT2‐3A* in transgenic cotton fibres at the secondary wall‐thickening stage. (a) Opened bolls with mature fibres; (b and c) DMACA staining of 11‐ and 27‐DPA ovules and fibres, respectively; (d) qRT–PCR analysis of *GhTT2‐3A* transcription during fibre development; (e) levels of soluble PAs in 11‐ and 27‐DPA fibres. **Indicates significant increase (*t*‐test, *P *<* *0.01) in PA content in transgenic fibres compared to the null segregant. Bars = 1 cm.

As earlier mentioned, no *GhTT2‐3A* overexpressing cotton plant was obtained, presumably because high levels of PAs inhibited cotton regeneration (Akagi *et al*., 2009). To verify the function of *GhTT2‐3A* in the brown pigmentation of cotton fibres, we employed a fibre‐specific promoter of the secondary wall‐thickening stage (*FbL2A*) (Rinehart *et al*., 1996) to direct the expression of *GhTT2‐3A* in transgenic cottons. Consequently, three independent *FbL2A:GhTT2‐3A* transgenic lines with moderately brown mature fibres were obtained (Figures 4a and S7). As expected, *GhTT2‐3A* was up‐regulated in the transgenic fibres preferentially after 16 DPA, and PAs significantly accumulated at 27 DPA, but not at 11 DPA (Figure 4b–e). These results indicate that up‐regulation of *GhTT2‐3A* in the fibres of the secondary wall‐thickening stage was effective and sufficient to induce PA biosynthesis and accumulation in fibres and to confer brown pigmentation to mature cotton fibres.

### GhTT2‐3A is capable of activating the whole PA pathway in cotton fibres

To further clarify the molecular mechanism underlying the activation of PA biosynthesis and accumulation in cotton fibres by GhTT2‐3A, we first investigated the transcriptional changes in PA structural genes in the transgenic fibres. In the developing fibres of *FbL2A:GhTT2‐3A* cottons, all the investigated genes are significantly up‐regulated in the 22‐DPA fibres compared with the null segregant (Figure S8), whereas their expressions are relatively very low in the 11‐DPA fibres, which coincides with the levels of *GhTT2‐3A* transcripts and soluble PAs. This result is in agreement with that of *GhTT2‐3A* overexpressing calli, confirming that the up‐regulation of *GhTT2‐3A* activates PA structural genes and promotes PA biosynthesis and brown pigmentation in cotton fibres.

Next, transcriptomic changes in 22‐DPA fibres from *FbL2A:GhTT2‐3A* cotton and its null segregant were analysed by RNA‐seq. Besides those PA synthase genes previously mentioned, a total of 170 significantly differentially expressed genes were identified, including 149 up‐regulated and 21 down‐regulated in *FbL2A:GhTT2‐3A* fibres compared to the control (Table S3). At least 62 up‐regulated genes are probably involved in PA biosynthesis and accumulation. These genes could be divided into four classes. The first class comprises 32 genes that encode PA synthases, including phenylalanine ammonia lyase to anthocyanidin reductase (He *et al*., 2008; Hinchliffe *et al*., 2016; Xiao *et al*., 2014). The second gene class encodes assistant proteins or enzymes that are essential to flavonoid synthases, including NADPH‐cytochrome P450 reductase, which is required in the oxidative reaction catalysed by C4H (Sundin *et al*., 2014), glutamine synthetase, which presumably functions in the recycling or relief from NH4^+^ toxicity caused by PAL (Guan *et al*., 2016) and CHI‐like proteins (Jiang *et al*., 2015). The third class includes transporters such as MATE protein TT12 (Zhao and Dixon, 2009), glutathione S‐transferase (TT19) (Pérez‐Díaz *et al*., 2016) and autoinhibited H^(+)^‐ATPase (TT13) (Appelhagen *et al*., 2015), which are involved in the transportation of PA monomers into vacuoles. The last class contains enzymes in pathways that lead to the generation of precursors of PA biosynthesis, that is the shikimate pathway to produce phenylalanine and enzymes to form malonate‐CoA (Chypre *et al*., 2012; Hinchliffe *et al*., 2016). Notably, among these differentially expressed genes, the flavonoid structural genes generally have highest expression levels in the *FbL2A:GhTT2‐3A* fibres and highest change folds of expression level compared to the control (Table S3), implying that these genes may be directly related to GhTT2‐3A and PA biosynthesis and accumulation.

**Figure 5:**
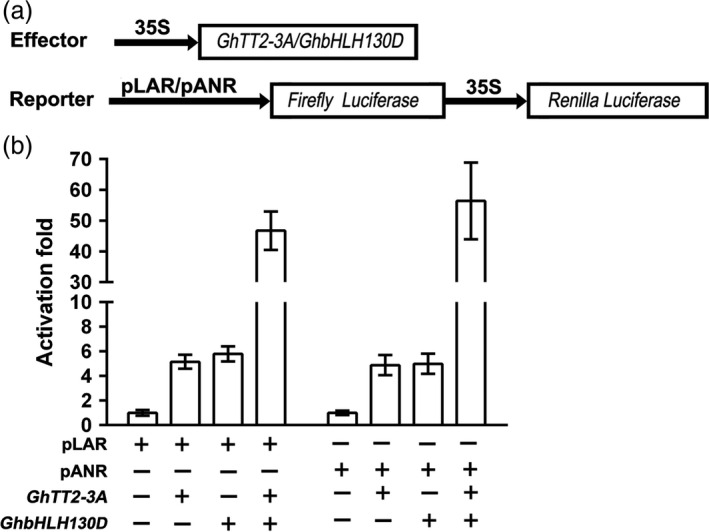
Synergistic activation of cotton *LAR* and *ANR* promoters by GhTT2‐3A and GhbHLH130D. (a) Schematic of the expression cassettes of effector and reporter vectors. (b) the activation effects on the promoters of LAR and ANR genes. The activator and *Renilla* luciferase genes are driven by CaMV35S promoter (35S). The promoter activities are presented as the activity ratio of firefly to *Renilla* luciferases.

In addition to *GhTT2‐3A* (*Gh_A07G2341*), RNA‐seq also showed that *Gh_D11G1273* (*GhbHLH130D*), a homolog of *Arabidopsis* PA regulator TT8, was significantly up‐regulated in *FbL2A:GhTT2‐3A* fibres (>4.5‐fold). To verify the functions of GhTT2‐3A and GhbHLH130D in regulating PA structural genes, dual‐luciferase assays were employed to detect their effects on the promoter activities of two PA‐specific synthase genes (*GhANR*,* Gh_A05G1424,* and *GhLAR*,* Gh_D12G1686*). Figure 5 shows that both GhTT2‐3A and GhbHLH130D individually activate the promoters of the *GhANR* and *GhLAR* genes to a moderate level (around fivefold), whereas co‐expression of these two genes dramatically increase the activation effects (around 50‐fold). These findings indicate that the regulation of the PA pathway in brown cotton fibres is similar to that in *Arabidopsis* seed coat; that is, R2R3‐MYB (GhTT2‐3A) and bHLH (GhbHLH130D) proteins activate downstream structural genes synergistically (Baudry *et al*., 2004; Koes *et al*., 2005) and consequently promote PA biosynthesis and accumulation in developing fibres.

### Characterization of mature *FbL2A*:*GhTT2‐3A* transgenic fibres

**Figure 6:**
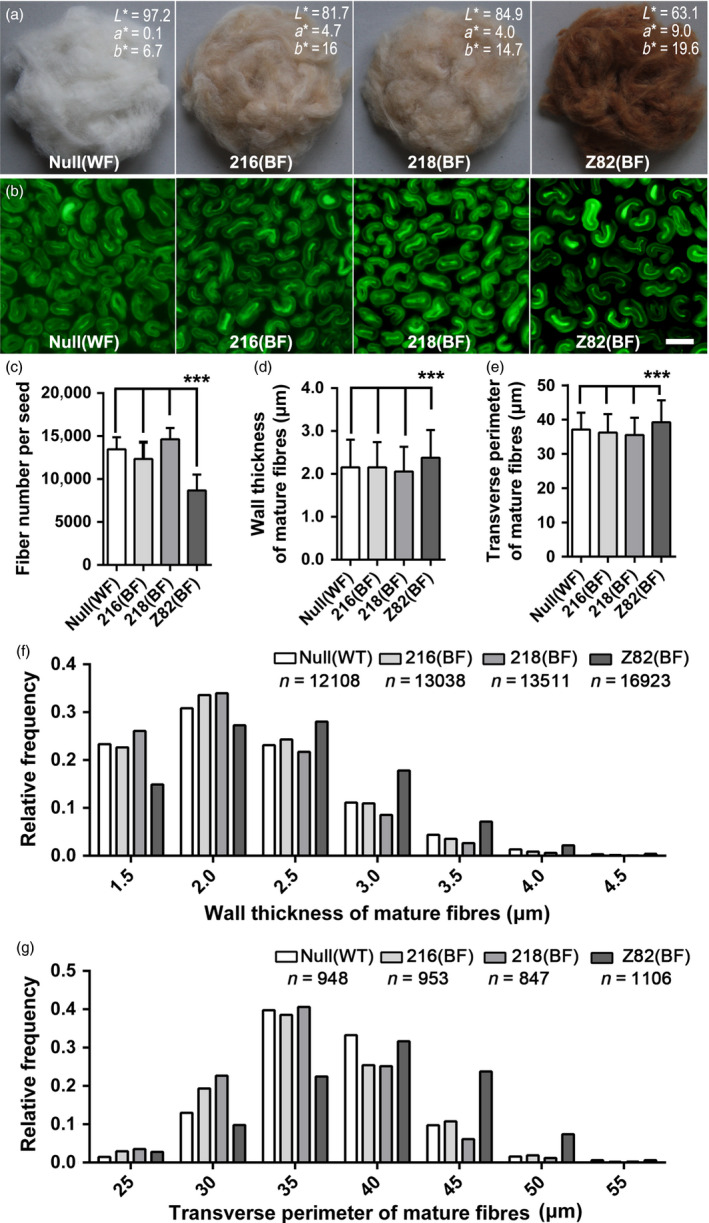
Characterization of mature *FbL2A:GhTT2‐3A* transgenic fibres. (a) The ginned mature fibres with the average CIE colour space values of 10 measurements (L*a*b*) indicated. L* indicates whiteness (0 =  black; 100 =  white); a* indicates colours from greenish (negative values) to reddish (positive values); and b* represents colours from bluish (negative values) to yellowish (positive values). (b) The micrographs of transverse sections of mature fibres. Bar = 20 μm. (c) Number of fibres per seed. (d) Wall thickness of mature fibres. (e) Transverse perimeter of mature fibres. (f and g) Frequency distributions of data in d and e, respectively. Error bars represent standard deviations, and ***indicates significant difference (*P *<* *0.0001), compared with *Lc1* control (Z82).

Brown coloration in mature fibres is generally linked to lower yield and inferior fibre quality (Efe *et al*., 2009; Feng *et al*., 2015; Hua *et al*., 2009; Zhang *et al*., 2009). To elucidate the influence of brown coloration in *FblA2:GhTT2‐3A* fibres on productivity and fibre quality, a randomized complete block experiment was performed to compare lint percentage and several fibre quality traits of *FbL2A:GhTT2‐3A* transformants to that of the white‐ and brown‐fibre controls. ANOVA and multiple comparisons demonstrate that *FbL2A:GhTT2‐3A* transgenic cottons (216 and 218) have no significant changes in lint percentage, fibre length, uniformity and strength compared to the white‐fibre null segregant, which exhibits superior traits relative to the *Lc1* brown‐fibre control (Tables 2 and S4). Microscopic observations indicate that, compared with *Lc1* control, *FbL2A:GhTT2‐3A* transgenic cottons and the null segregant have significantly increased fibre numbers per seed, and their mature fibres have significantly thinner walls and smaller transverse perimeters, while these parameters do not vary significantly between *FbL2A:GhTT2‐3A* transgenic cottons and the null segregant (Figure 6). These data indicate that specific up‐regulation of *GhTT2‐3A* in the secondary wall‐thickening fibres confers brown coloration to mature fibres, but without affecting fibre quality and lint percentage.

**Table 2:** Lint percentage and fibre quality of *FbL2A:GhTT2‐3A* cottons (216 and 218), white‐ (null)‐ and brown‐fibre (Z82) controls

Genotypes	Lint percentage (%)	Fibre length (mm)	Fibre uniformity (%)	Fibre strength (cN.tex^−1^)
Null (WF)	38.51 ± 1.32^A^	30.31 ± 0.67^A^	86.08 ± 0.68^A^	30.62 ± 0.81^A^
216 (BF)	36.39 ± 0.96^A^	30.31 ± 0.66^A^	85.76 ± 1.12^A^	30.82 ± 1.45^A^
218 (BF)	36.13 ± 0.81^A^	30.40 ± 1.14^A^	85.76 ± 1.22^A^	30.13 ± 2.03^A^
Z82 (BF)	28.62 ± 1.89^B^	22.97 ± 0.94^B^	79.54 ± 0.95^B^	24.08 ± 0.60^B^

The averages ± SD of six plot values (three replicates and two harvest times) are indicated. For each trait, the identical and different upper‐case letters show nonsignificant and significant differences (LSD, *P *<* *0.01) between materials, respectively.

## Discussion

### *GhTT2‐3A* is the gene that controls brown coloration in cotton fibres

The major objective of this study was to identify the gene that controls brown coloration in cotton fibres and to determine its potential for improving transgenic NCCs. On the basis of previous reports (Hinchliffe *et al*., 2016; Wang *et al*., 2014; Zhang *et al*., 2009), the brown fibre gene (*Lc1*) was mapped to a region of around 67 kb in length that included three protein‐coding genes (Figure 3), among which only *GhTT2‐3A* was significantly expressed in developing brown fibres but not in white fibres (Figures S4 and S5). Moreover, up‐regulation of *GhTT2‐3A* rescued PA synthesis and accumulation in *FbL2A:GhTT2‐3A* transgenic fibres and resulted in brown pigmentation in mature cotton fibres (Figures 4 and S7). These findings collectively indicate that *GhTT2‐3A* is the gene controlling brown coloration in *Lc1* cotton fibres. Considering that the brown‐ and white‐fibre materials (T586 and Yumian No. 1) have identical coding regions for *GhTT2‐3A*, the *Lc1* phenotypes should be attributed the up‐regulation of *GhTT2‐3A* in developing fibres (Figures S3 and S4) (Hinchliffe *et al*., 2016).

**Figure 7:**
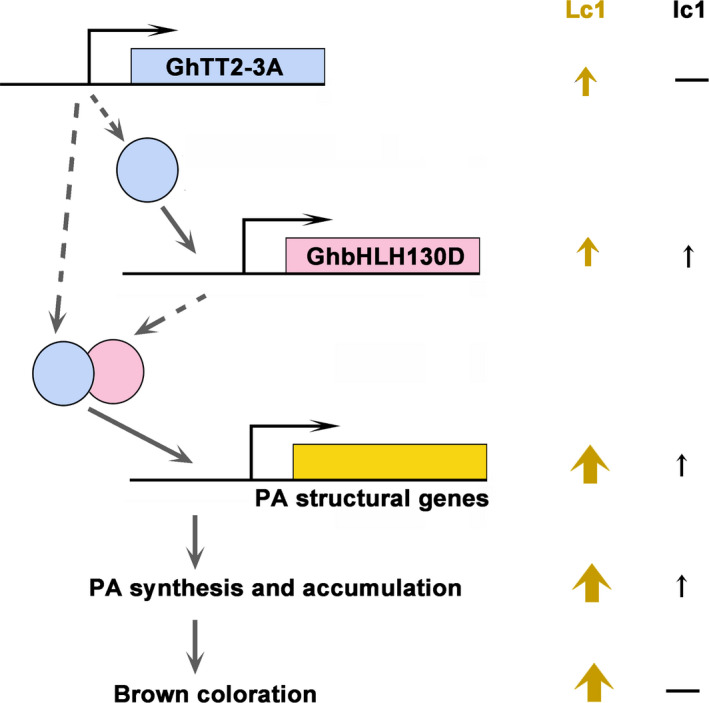
The regulatory model controlling brown coloration in cotton fibres by *GhTT2‐3A*. Coding genes are depicted as bars with line and arrow showing promoter and transcription direction, respectively. Proteins are represented with circles filled with identical colour as coding genes, which linked by dashed arrows. Solid arrows suggest activation or promotion effects on the next events. Upright arrows and dashes (—) indicate the presence and absence of certain event, respectively, and arrow sizes indicate approximately the levels of gene transcription, PA accumulation or brown coloration in the brown (Lc1) and white (lc1) fibres.

Several reports have suggested that brown pigments belong to PAs or their derivatives (Feng *et al*., 2014; Hinchliffe *et al*., 2016; Xiao *et al*., 2007, 2014). In *Arabidopsis*, the PA pathway is transcriptionally regulated by a tripartite complex consisting of TT2, TT8 and TTG1, where TT8 is transcriptionally activated by TT2 and acts synergistically with TT2 to enhance the transcription of PA structural genes (Baudry *et al*., 2004, 2006; He *et al*., 2008; Koes *et al*., 2005; Xu *et al*., 2014). Similarly, the results of the present study show that up‐regulation of *GhTT2‐3A* significantly promotes the expression of *GhbHLH130D* (a TT8 homolog) (Yan *et al*., 2015), and these two transcription factors synergistically activate the promoters of two PA structural genes (*GhANR* and *GhLAR*; Figure 5). Based on the transcriptional regulation of the PA pathway in model plants, we propose that elevated expression of *GhTT2‐3A* in *Lc1* fibres enhances *GhbHLH130D* expression and dramatically promotes PA synthesis and accumulation and brown pigmentation by up‐regulating PA structural genes by the synergistic action of GhTT2‐3A and GhbHLH130D (Figure 7). Notably, the synergism with GhbHLH130D further enhances the transcriptional activation effects of GhTT2‐3A on PA structural gene expression, and the extent of up‐regulation of PA structural genes is generally higher than that of *GhbHLH130D* in brown cotton fibres (Table S3).

Dark‐brown fibre (*Lc1*) is a traditional genetic marker in cotton (Kohel, 1985; Zhang *et al*., 2005). Recently, Hinchliffe *et al*. (2016) reported the up‐regulation of *GhTT2_A07* (*GhTT2‐3A*) in *Lc1* fibres and suggested that a co‐segregated genomic inversion of a 1.4‐Mb segment upstream of this gene is the causative mutation of *Lc1*. Similarly, we identified a 2110‐bp inverted fragment in the *GhTT2‐3A* BAC clone (BAC‐TT2‐3A), which was 13 086 bp upstream of the initial ATG of *GhTT2‐3A*. Besides the genomic inversion, we identified two differentially inserted retrotransposons by comparing the upstream sequences of *GhTT2‐3A* of T586 and TM‐1 (Figures 3 and S9a). Further investigation using different brown‐ and white‐fibre materials showed that none of these three sequence divergences co‐segregated with fibre coloration (Figure S9b), suggesting that these sequence variations may not be directly related to the *Lc1* phenotype and the causative factor leading to the high‐level expression of *GhTT2‐3A* in *Lc1* fibres remains unknown.

### Biotechnological strategies to improve NCCs

Limited colour diversity and inferior yield and fibre quality have been the major obstacles to the development of the NCC industry (Efe *et al*., 2009; Feng *et al*., 2015; Hua *et al*., 2009; Kimmel and Day, 2001; Zhang *et al*., 2009). Although numerous efforts have been taken to introduce new colour to cotton fibres, to our knowledge, no successful attempts to introduce brown mature fibres into white‐fibre acceptors have been reported to date. Furthermore, our results indicate that the negative effects of coloration on fibre development could be largely eliminated by specifically activating PA synthesis and accumulation in the fibres of the secondary wall‐thickening stage. Notably, the brown colour of transgenic *FbL2A:GhTT2‐3A* cotton fibres is not as dark as that of *Lc1* cottons. We attribute the light fibre coloration to the lower PA contents in the transgenic fibres compared to that in *Lc1* fibres (Figures 4 and S5). In an attempt to obtain darker fibre colours, we plan to co‐express *GhbHLH130D* and *GhTT2‐3A* in fibres during secondary wall thickening (Figures 5 and 7).

Proanthocyanidin is generally synthesized and accumulated in a tissue‐specific or inducible manner (Akagi *et al*., 2009; Debeaujon *et al*., 2003; Li *et al*., 2016a; Liu *et al*., 2014; Mellway *et al*., 2009). The constitutively activated PA pathway inhibits plant growth and development (Akagi *et al*., 2009). Previously, an intermediate of PA biosynthesis (naringenin) was reported to retard cotton fibre cell elongation (Tan *et al*., 2013), which partially explained the negative correlation between brown coloration and fibre quality and yield. The findings of the present study did not observe any significant changes in the structure and quality of *FbL2A:GhTT2‐3A* fibres compared to the wild‐type control (Table 2 and Figure 6), suggesting that up‐regulation of the PA pathway and PA accumulation in the fibres of secondary wall‐thickening stage might have a lesser effect, if any, on cotton fibre development and final quality. This implied that the secondary wall‐thickening stage might be an appropriate period to manipulate cotton colour via the biosynthesis and accumulation of PAs and other flavonoids. Moreover, when considering strategies to biotechnologically improve NCCs via other natural pigments, it is essential to elucidate their physiological influence on fibres at different developmental stages.

## Experimental procedures

### Cloning and sequence analysis of TT2 homologous genes

A total of 47 putative homologs of *Arabidopsis* TT2 in the *G. raimondii* genome were downloaded from Phytozome 12.1 (http://www.phytozome.net/cotton.php) (Goodstein *et al*., 2012). These sequences and TT2 were employed to construct a neighbour‐joining (NJ) tree with 1000 replicates for bootstrap analysis in MEGA 6.0 (Tamura *et al*., 2013). The proteins clustered with TT2 were regarded as TT2 homologs, and their homologous genes were further identified by tBLASTN searching the assembled *G. arboreum* (http://www.cottongen.org) and *G. hirsutum* (https://phytozome.jgi.doe.gov/pz/portal.html#!info?alias=Org_Ghirsutum_er) genomes using a stand‐alone software. Multiple alignments were all performed with ClustalW method in MEGA 6.0 (Tamura *et al*., 2013).

The coding regions of cotton TT2 homologs in *G. raimondii, G. arboreum* and *G. hirsutum* (brown‐fibre line T586 and white‐fibre line Yumian No. 1) were then amplified using primers designed according to the *G. raimondii* homologs (Table S5). PCRs were performed using PrimeSTAR^®^ Max DNA polymerase (TaKaRa, Dalian, China) according to the manufacturer's instructions. All PCR products were cloned into pGEM‐T Easy vectors (Promega) and sequenced in BGI (Shenzhen, China). All these sequences were deposited in GenBank as Accession Nos. MG431343–MG431368.

### RNA extraction and quantitative RT–PCR analyses

Total RNAs were extracted from various cotton tissues using a rapid plant RNA extraction kit (Aidlab, Beijing, China), and genomic DNA degradation and first‐strand cDNA synthesis were performed using a PrimeScript™ RT reagent kit with gDNA eraser (TaKaRa, Dalian, China). Quantitative PCRs were performed in a CFX96 real‐time PCR system (Bio‐Rad, CA) using SYBR Green Supermix. The thermocycling parameters were as follows: 95 °C for 2 min, followed by 40 cycles of 95 °C for 10 s, 57 °C for 20 s and a standard melting curve to monitor PCR specificity. *GhACT4* and *GhUBQ14* were used as reference to normalize the transcript levels of target genes (Artico *et al*., 2010). The PCR results were analysed using Bio‐Rad CFX Manager 2.0 provided by the manufacturer (Bio‐Rad). Primers used for quantitative PCRs are listed in Table S6.

### Vector construction and cotton transformation

A modified pBI121 vector p5 (pBI121‐GN) containing selection marker *NPTII* and *GUS* genes was used to construct plant expression vectors (Luo *et al*., 2007). The cDNA sequences of *GhTT2s* (*GhTT2‐1D*,* GhTT2‐2D*,* GhTT2‐3A*,* GhTT2‐4D* and *GhTT2‐5D*) ORFs were amplified from the appropriate T586 tissues. After cloning in the pGEM‐T Easy vector (Promega) and sequencing, the ORFs were excised using *Bam*HI and *Eco*RI, and inserted downstream to a CaMV35S promoter in the p5 vector that was restricted by the same enzymes, resulting in the overexpression vectors of *GhTT2s*. To construct fibre‐specific expression vectors, the promoter of *FbL2A* gene that was specifically expressed in fibres of the secondary wall‐thickening stage (Rinehart *et al*., 1996) was amplified from sea island cotton. The promoter was constructed upstream to *GhTT2‐3A* by replacing the CaMV35S promoter in the *GhTT2‐3A* overexpression vector using *Hin*dIII and *Bam*HI sites that were introduced by PCR (Table S5). All these expression vectors were transferred into *Agrobacterium tumefaciens* strains (LBA4404), and the resulting *Agrobacterium* strains were used to transform a white‐fibre cultivar Jimian No. 14 (J14) as previously described (Luo *et al*., 2007).

Southern blot of the T0 generation of *FbL2A:GhTT2‐3A* transformants was performed as previously described (Zhang *et al*., 2011), using a *GUS* fragment amplified from the vector p5 as probe. The genomic DNAs were restricted by *Hin*dIII, and a DIG‐High Prime DNA Labeling and Detection Starter Kit (Roche) was employed in preparing DIG‐labelled probes and to detect hybridization signals. The presence and expression of transgenes in all generations were monitored by GUS staining and quantitative RT–PCR.

### Genetic mapping of *Lc1*

The BAC libraries of brown‐fibre cotton line T586 and white‐fibre line Yumian No. 1 were constructed using the pIndigoBAC‐5 vector (Epicentre Inc., Madison, WI) by Cosete Technology (Jinan, China). Two BAC clones containing *GhTT2‐2A* and *GhTT2‐3A* (GenBank Accession Nos. MG431369 and MG431370, respectively) were screened with gene‐specific primers via a PCR‐based method (Yim *et al*., 2007) and sequenced by shotgun strategy at BGI (Shenzhen, China). According to these BACs and their surrounding sequences in *G. raimondii* genome (Paterson *et al*., 2012), over 20 fragments were amplified from T586 and white‐fibre variety Yumian No. 1 and compared. Polymorphic loci were then further amplified with subgenome‐specific primers (Table S1). SSR and Indel loci were amplified and detected as described elsewhere (Zhang *et al*., 2005, 2009). SNPs were genotyped via high resolution melting (HRM) method, which was performed in a CFX96 real‐time PCR system using a Precision Melt Supermix (Bio‐Rad). The HRM data were analysed using Precision Melt Analysis Software 1.2 (Bio‐Rad).

Two segregation populations were used to map the *Lc1* gene. The RIL population, including 270 F_2:7_ lines, was derived from T586 × Yumian No. 1 as previously reported (Zhang *et al*., 2005, 2009). The enlarged F2 population, derived from the hybrid between a brown‐fibre RIL (RIL152) and white‐fibre parent Yumian No. 1, contained 1698 individuals. The populations were grown in the field, and fibre colour phenotype of each individual was scored by DMACA staining at 20 DPA and visual inspection at maturation. Genomic DNAs were extracted using a plant DNA extraction kit (Aidlab, Beijing, China). Linkage analysis and graphic presentation of linkage groups were conducted as described elsewhere (Zhang *et al*., 2009).

### Detection and quantification of PAs

DMACA staining was employed to visualize PAs in various cotton tissues (Xiao *et al*., 2007). The soluble PAs in cotton tissues were extracted and quantified as described elsewhere (Pang *et al*., 2008). Briefly, approximately 0.5 g of fresh cotton tissues was ground to a fine powder in liquid nitrogen with a mortar and pestle and extracted twice in 1 mL of 80% acetone containing 1% ascorbate. The soluble PAs in the supernatants were detected spectrophotometrically at the wavelength of 640 nm after reacting with DMACA, and PA content was calculated with (+)‐catechin as standard. The extractions were performed in triplicate.

### Transcriptomic analysis

Total RNAs were extracted from 22‐DPA fibres of *FbL2A::GhTT2‐3A* cotton (Line 216), and its null segregant was used for transcriptomic analysis. RNA detection, sequencing and routine data analysis were performed by Novogene (Beijing, China). Raw data were deposited in GenBank (SAR: PRJNA416219). Paired‐end clean reads of 125 nt in size (over 3.3 Gb) were aligned to the annotated genome of *G. hirsutum* (Zhang *et al*., 2015) using TopHat v2.0.12 (Trapnell *et al*., 2009). HTSeq v0.6.1 was used to count the reads mapped to each gene, and then, number of fragments per kilobase of transcript sequence per million base pairs sequenced (FPKM) of each gene was calculated (Anders and Huber, 2010). After the read counts were adjusted by the edgeR program package using a scaling normalized factor (Robinson *et al*., 2010), differential expression analysis of *FbL2A::GhTT2‐3A* and wild‐type fibres was performed using the DEGSeq R package (1.20.0). A corrected *P*‐value of 0.005 and log_2_ (fold change) of 1 were set as the threshold for significant differential expression.

### Dual‐luciferase assay

Promoter sequences of approximately 2 kb upstream of the initial ATGs of *GhANR* (*Gh_A05G1424*) and *GhLAR* (*Gh_D12G1686*) were amplified from T586 and inserted into the *Hin*dIII and *Bam*HI sites of pGreen 0800‐LUC (Espley *et al*., 2007) to generate reporter vectors. To construct the overexpression vector for *GhbHLH130D*, the ORF was amplified from cDNAs of 20‐DPA brown fibres and constructed into the *Bam*HI and *Eco*RI sites of p5. These constructs, along with the empty p5 vector, were transformed into *A. tumefaciens* strain GV3101. *A. tumefaciens* cells harbouring the effector and reporter vectors were infiltrated into *Nicotana benthamiana* leaves as described elsewhere (Shan *et al*., 2014). Leaf discs of approximately 1.5 cm^2^ in size were sampled by punching and homogenized in 200 μL of an extraction buffer (1 mm dithiothreitol, 0.1 m phosphate buffer, pH 8.0). The luciferase activity of 50 μL supernatants was assayed in an automatic microplate reader (Infinite M200 Pro, Tecan, Switzerland) using a Dual‐Glo Luciferase Assay System (Promega). For each treatment, two technical repeats of three biological replicates were detected. The activation effects were expressed as the ratio of firefly to *Renilla* luciferase activity.

### Fibre analysis

A randomized complete block experiment was performed to compare fibre traits of homozygous T4 generation *FblA2:GhTT2‐3A* cottons (216 and 218), a null segregant and an *Lc1* line Z82, which was a homozygous brown‐fibre BC5 line derived from a cross of T586 × J14 with J14 as the recurring parent. The experiment using three replicates was conducted in the experimental farm of Southwest University (Chongqing, China) in 2016. Each plot contained 30 plants grown in three rows with 1‐m spacing as described elsewhere (Zhang *et al*., 2011). For each plot, mature seed fibres were collected from naturally opened bolls at two time points (1 September 2016 and 21 September 2016). After drying and ginning using a roller gin (SY‐20, Jianghe Machinery Plant, Xinxiang, Henan, China), fibres and cottonseeds were separately weighed to determine lint percentages. Fibres from each plot at specified harvest time were randomly sampled for three duplicates and subjected to fibre quality measurements at a HVI system (HFT 9000, Uster Technologies, Swiss) in the Center of Cotton Fiber Quality Inspection and Testing, Chinese Ministry of Agriculture (Anyang, Henan, China). The plot values for fibre quality traits (fibre length, fibre uniformity and fibre strength) at certain harvest time were the average of three duplicates and used in statistical analysis. The final data included the values for lint percentage and three fibre quality traits from three replicates of four lines and two harvest times. For each trait, two‐way ANOVA and subsequent multiple comparison were performed using intrinsic programs in GraphPad Prism software (v6.01, http://www.graphpad.com/scientific-software/prism/).

### Microscopic observation of mature fibres

For microscopic observation of the mature fibres, *FblA2:GhTT2‐3A* lines (216 and 218), a null segregant and the *Lc1* line Z82 were grown side by side in the field. Naturally opened bolls were harvested from the first nodes on the third to fifth fruit branches on the same day. Only the six cottonseeds in the middle of locule were collected for microscopic analysis. The fibres from the middle of cottonseed were fixed for transverse sectioning as described elsewhere (Li *et al*., 2016b). The sections were observed and imaged on a phase‐contrast microscope (OLYMPUS IX81). Wall thickness and perimeter of fibre transverse sections were measured in the collected images using ImageJ (http://imagej.net/Fiji). For each line, at least 100 fibre bundles from 10 different plants were observed, and over 6000 and 800 fibre sections were measured independently for fibre wall thickness and transverse perimeter, respectively. Lint number per seed was determined as previously described (Zhang *et al*., 2011). Briefly, fibres from 20 random cottonseeds were separated by hand and weighed (W1) on an analytical balance (XS105 Dual Range, METTLER). Six bundles of fibres (each around 1–2 mg) were randomly selected, teased and weighed (W2). The fibre bundles were fixed in a microcentrifuge tube and treated in boiling water for 10 min. Three segments were cut from the middle region of fibre bundles (the cutting length: ~1–2 mm). Each segment was separated in six drops of 45% (v/v) acetic acid, and the fibre snippet number was counted under a stereomicroscope (MVX‐10, OLYMPUS). The average of snippet numbers of the 18 segments (three segments × six bundles) was recorded as fibre number in a bundle (N2). The number of fibres per seed (N1) was calculated using the equation: N1 = (W1/20)/(W2/N2). For each line, the measurement was repeated for six times using the selected cottonseeds earlier described. Statistical analyses of these data, including one‐way ANOVA, multiple comparisons and frequency distribution, were performed with GraphPad Prism (v6.01, http://www.graphpad.com/scientific-software/prism/).

Fibres ginned using a roller gin (SY‐20, Jianghe Machinery Plant, Xinxiang, Henan, China) were randomly sampled for colour space measurement as described elsewhere (Hinchliffe *et al*., 2016).

## Supporting information


**Figure S1** Phylogenetic analysis of *G. raimondii* proteins similar to TT2.
**Figure S2** Alignment of TT2 and its homologs identified in *G. raimondii*.
**Figure S3** Structures of TT2 homologous genes from *G. arboreum*,* G. raimondii* and *G. hirsutum*.
**Figure S4** qRT‐PCR analyses of the expression of cotton TT2 homologous genes in brown‐ and white‐fiber cottons.
**Figure S5** Transcript levels of TT2 homologous genes and PA contents in brown and white fibers of different developmental stages.
**Figure S6** TT2 homologs promote PA biosynthesis and accumulation in transgenic cotton calli.
**Figure S7** Characterization of *FbL2A:GhTT2‐3A* transgenic cottons.
**Figure S8** qRT‐PCR analysis of PA structural genes in *FbL2A:GhTT2‐3A* transgenic fibers.
**Figure S9** Divergence of *GhTT2‐3A* upstream sequence between brown‐ and white‐fiber materials.

**Table S1** Markers used for fine mapping of *Lc1*.
**Table S2** Protein‐coding genes annotated in *Lc1* region.
**Table S4** Two‐way ANOVA in lint percentage and fiber quality traits of *FbL2A:GhTT2‐3A* fibers and controls from two harvest times.
**Table S5** Primers used in cloning.
**Table S6** Primers used in qRT‐PCR analysis.

**Table S3** Differentially expressed genes in 22‐DPA *FbL2A:GhTT2‐3A* fibers compared to the null segregant.
