# Supplementary material for: Up‐regulation of GhTT2‐3A in cotton fibres during secondary wall thickening results in brown fibres with improved quality
Source: Plant Biotechnol J. 2018 Apr 2;16(10):1735–47. doi: 10.1111/pbi.12910 (PMC6131414; doi:10.1111/pbi.12910)
Supplement: Supplementary file 1 — Figure S1 Phylogenetic analysis of G. raimondii proteins similar to TT2. Figure S2 Alignment of TT2 and its homologs identified in G. raimondii. Figure S3 Structures of TT2 homologous genes from G. arboreum, G. raimondii and G. hirsutum. Figure S4 qRT‐PCR analyses of the expression of cotton TT2 homologous genes in brown‐ and white‐fiber cottons. Figure S5 Transcript levels of TT2 homologous genes and PA contents in brown and white fibers of different developmental stages. Figure S6 TT2 homologs promote PA biosynthesis and accumulation in transgenic cotton calli. Figure S7 Characterization of FbL2A:GhTT2‐3A transgenic cottons. Figure S8 qRT‐PCR analysis of PA structural genes in FbL2A:GhTT2‐3A transgenic fibers. Figure S9 Divergence of GhTT2‐3A upstream sequence between brown‐ and white‐fiber materials. [file PBI-16-1735-s002.docx]

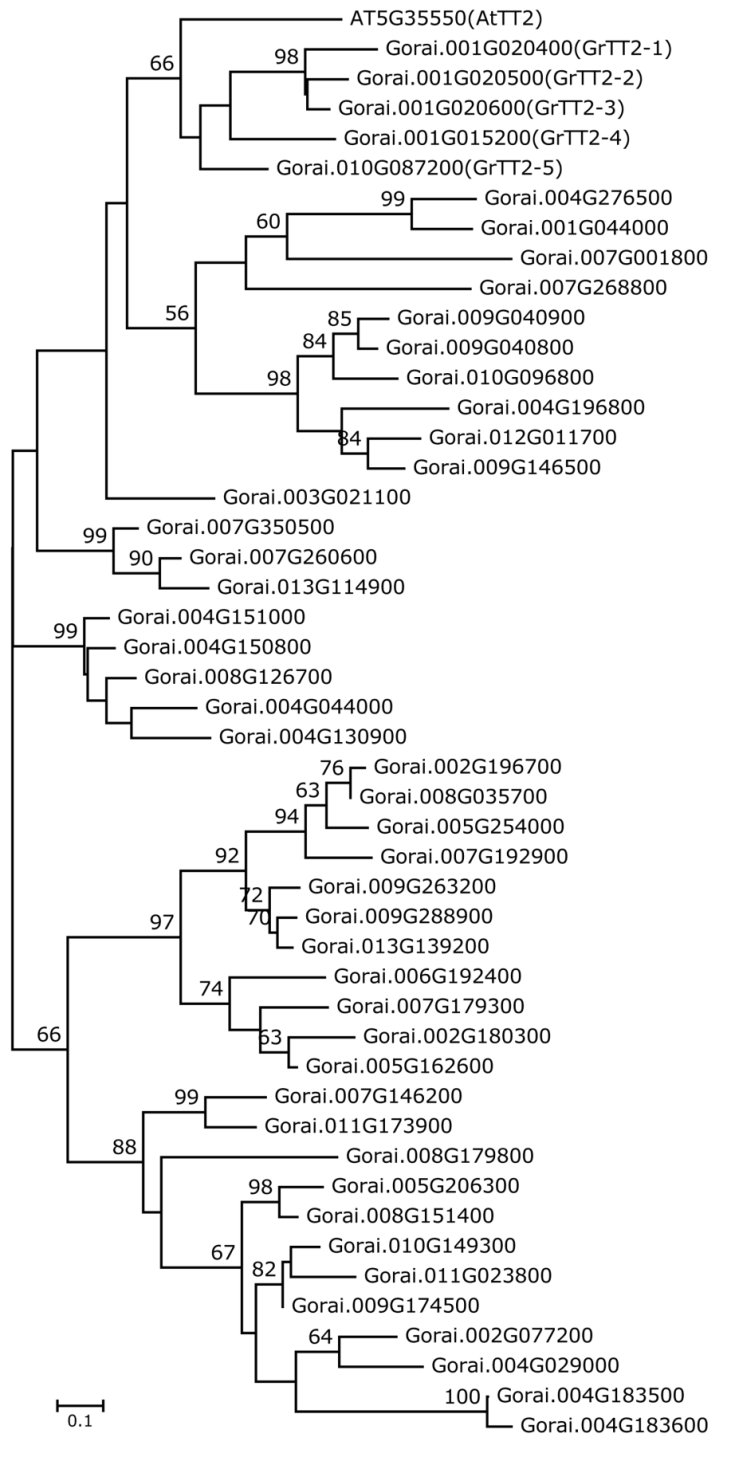


**Figure S1. Phylogenetic analysis of *G. raimondii* proteins similar to TT2**

The evolutionary history was inferred by using the Maximum Likelihood method in MEGA6 based on the JTT matrix-based model. The tree with the highest log likelihood (-6678.9336) is shown. The percentage of trees in which the associated taxa clustered together is shown above the branches (numbers less than 50 are omitted). The tree is drawn to scale, with branch lengths measured in the number of substitutions per site. The analysis involved Arabidopsis TT2 and 47 similar proteins from *G. raimondii*. All positions with less than 95% site coverage were eliminated.


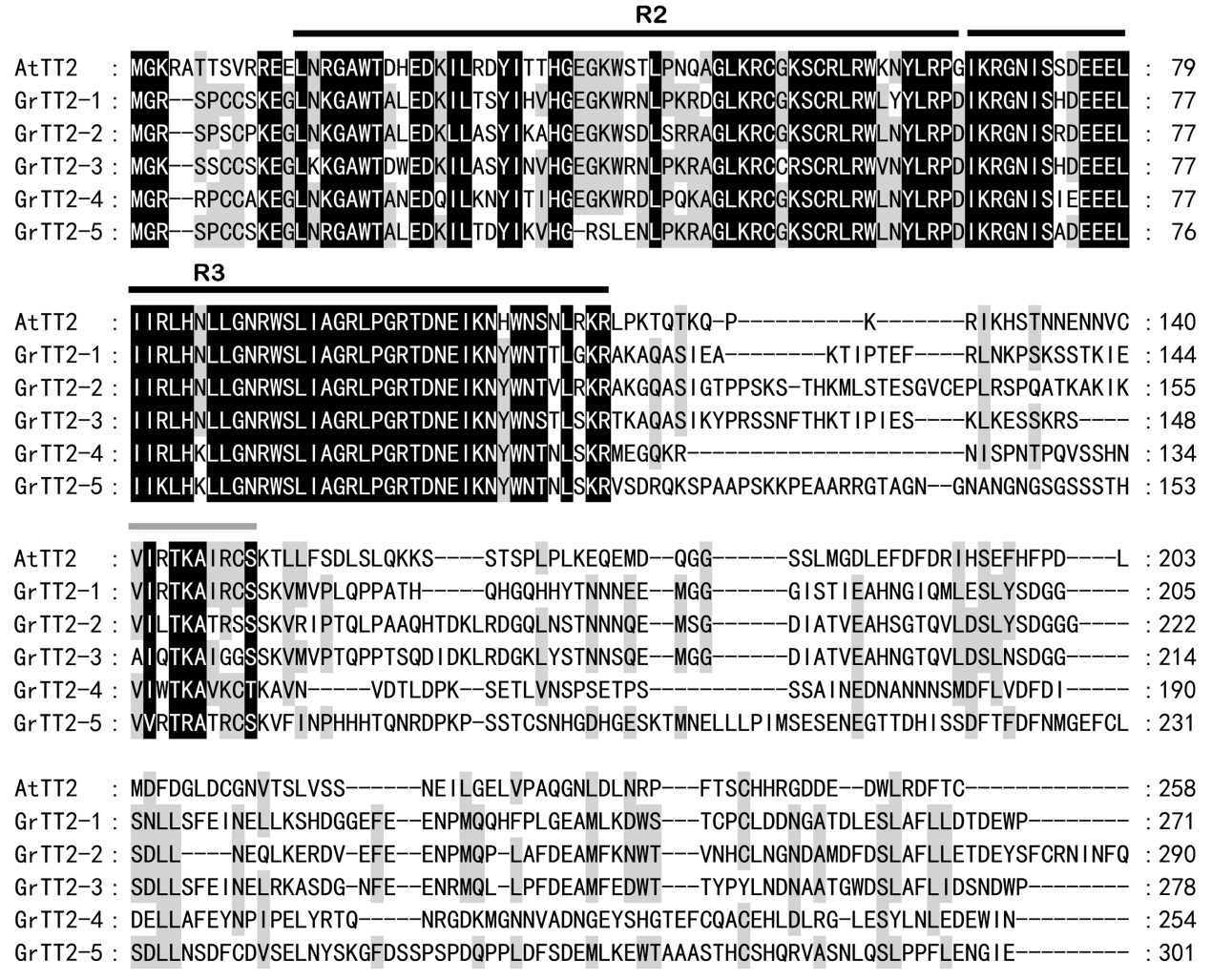


**Fugure S2. Alignment of TT2 and its homologs identified in *G. raimondii*.**

Amino acid residuals conserved in all or > 60% sequences are shaded in black and grey, respectively. Grey bar indicates the IRTKA[IL]RC[SN] motif found in TT2, and the black bars indicate the R2 and R3 repeats of the MYB domain.


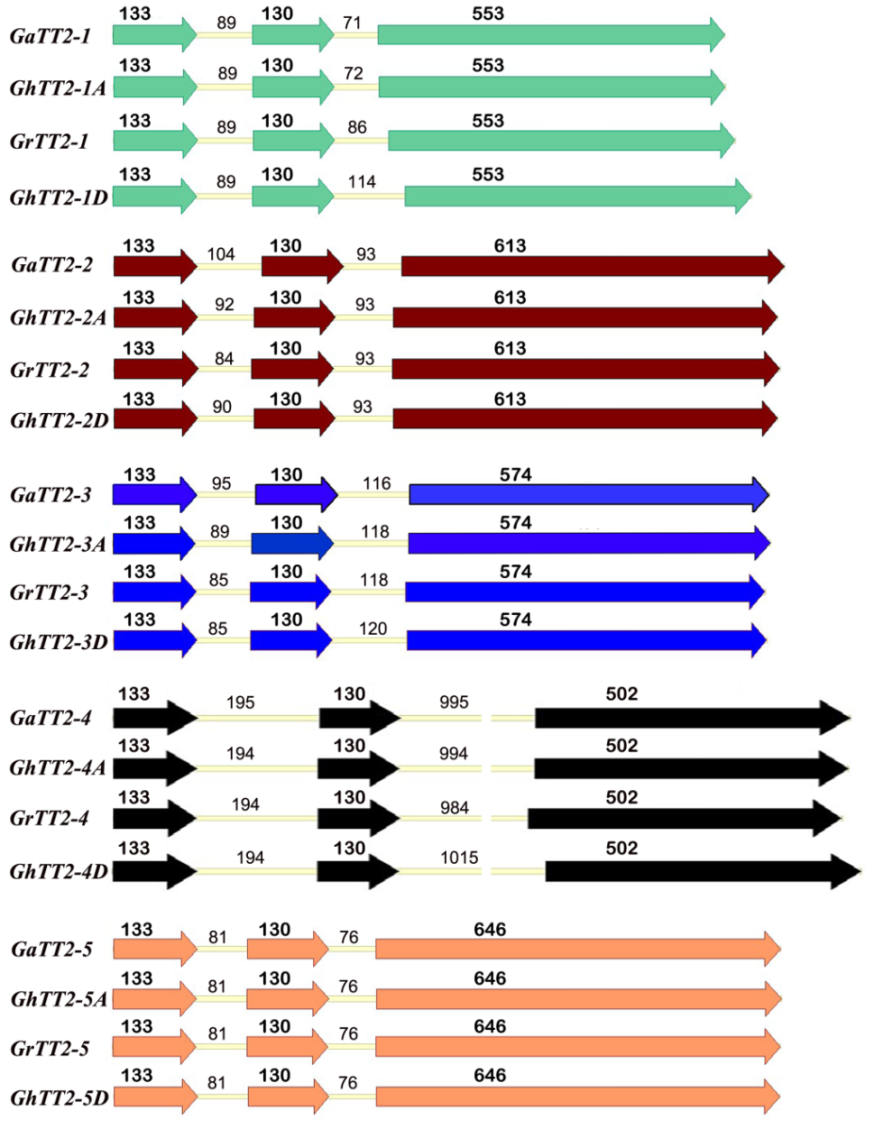


**Figure S3. Structures of TT2 homologous genes from *G. arboreum*, *G. raimondii* and *G. hirsutum***

Four genes in each homeologous group are aligned togather. The coding sequences are drawn in scale with length in bp indicated above exons (arrow) and introns (thin bars). Identical fragments in the 2^nd^ intron of *GoTT2-4*s are omitted for concision.


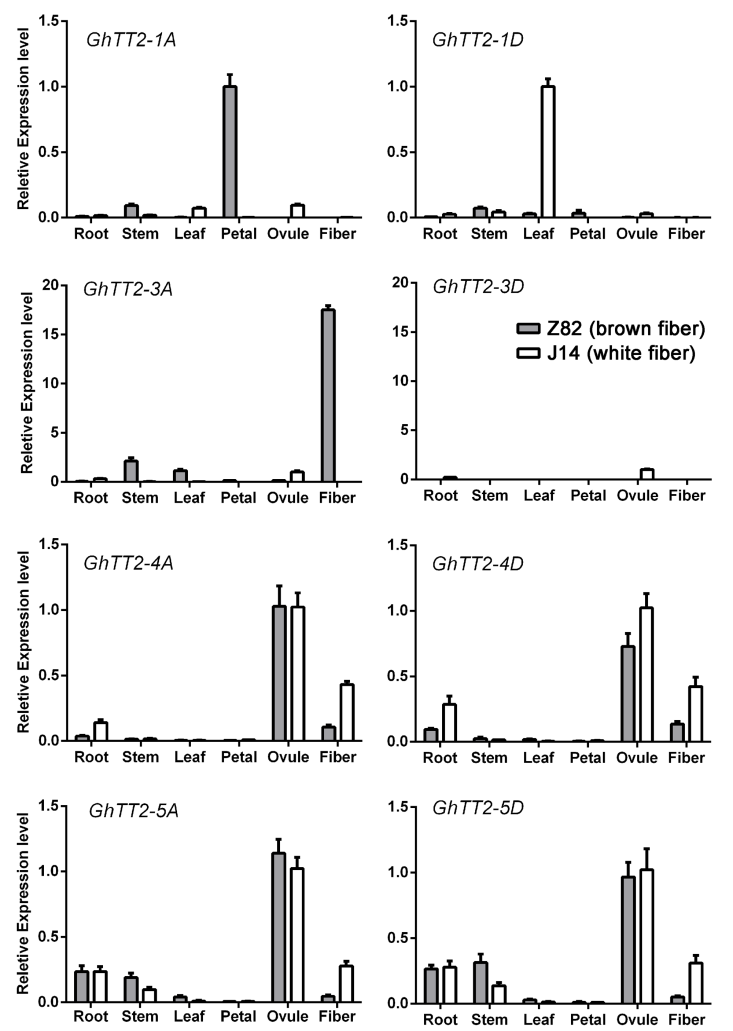


**Figure S4.** **qRT-PCR analyses of the expression of cotton TT2 homologous genes in brown- and white-fiber cottons**

RNAs are from roots, stems, leaves, petals, 10-DPA ovules and fibers in brown- and white-fiber cottons (Z82 and J14, respectively). Two genes (*GhTT2-2A* and *D*) are omitted for their very low expression levels.


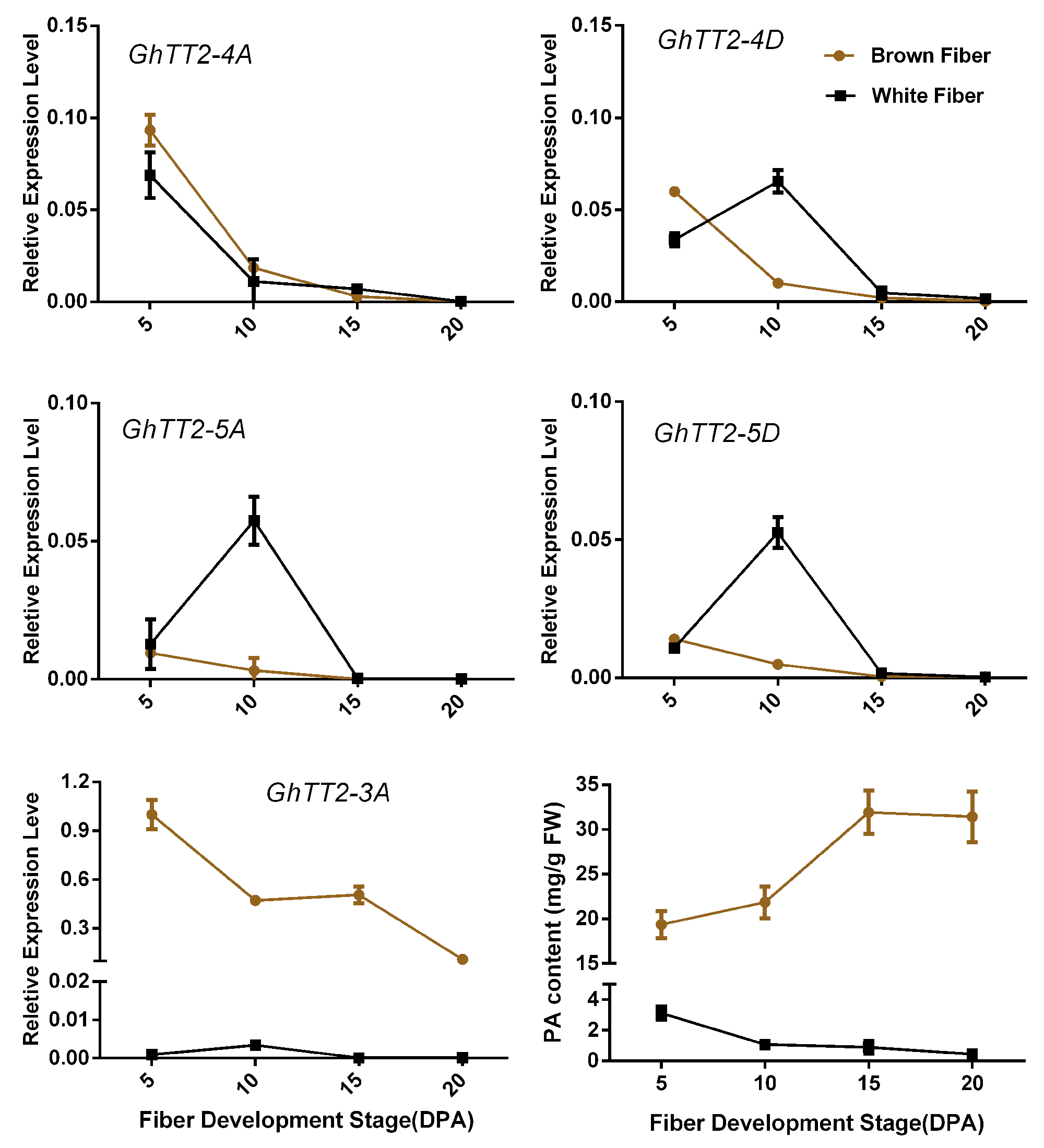


**Figure S5. Transcript levels of TT2 homologous genes and PA contents in brown and white fibers of different developmental stages**

RNAs and soluble PAs are extracted from fibers of 5, 10, 15 and 20 DPA in brown- and white-fiber cottons (Z82 and J14, respectively). Only the homologous genes with significant expressions in fibers are presented.


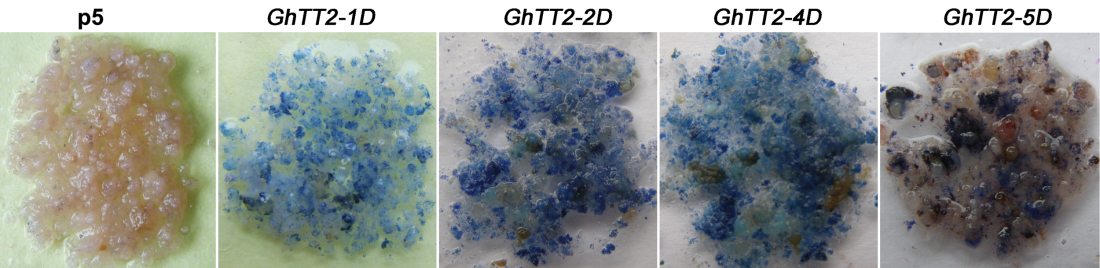


**Figure S6. TT2 homologs promote PA biosynthesis and accumulation in transgenic cotton calli**

The TT2 homologous genes (*GhTT2-1D*, *2D*, *4D* and *5D*) are constructed downstream to the constitutive promoter CaMV35S in plant expression vector p5, and transformed into J14 calli. The empty vector p5 was transformed in parallel as control.

**
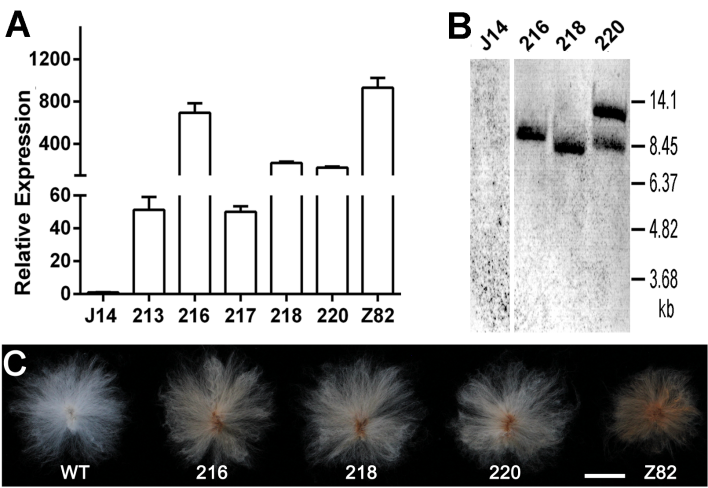
**

**Figure S7. Characterization of *Fbl2A:GhTT2-3A* transgenic cottons**

A, qRT-PCR analysis of the expression of GhTT2-3A in fibers of 20DPA. J14, the untransformed acceptor cotton; 213-220, transgenic cottons; Z82, a brown-fiber line with J14 background and *Lc1* from T586. B, Southern blotting of transgenic cottons. The total genomic DNAs were digested with *Hin*dⅢ, and hybrided to a DIG-labelled GUS fragment. C, Mature seed with fibers. WT, a null segregant of transgenic line 216. Bar = 1cm.


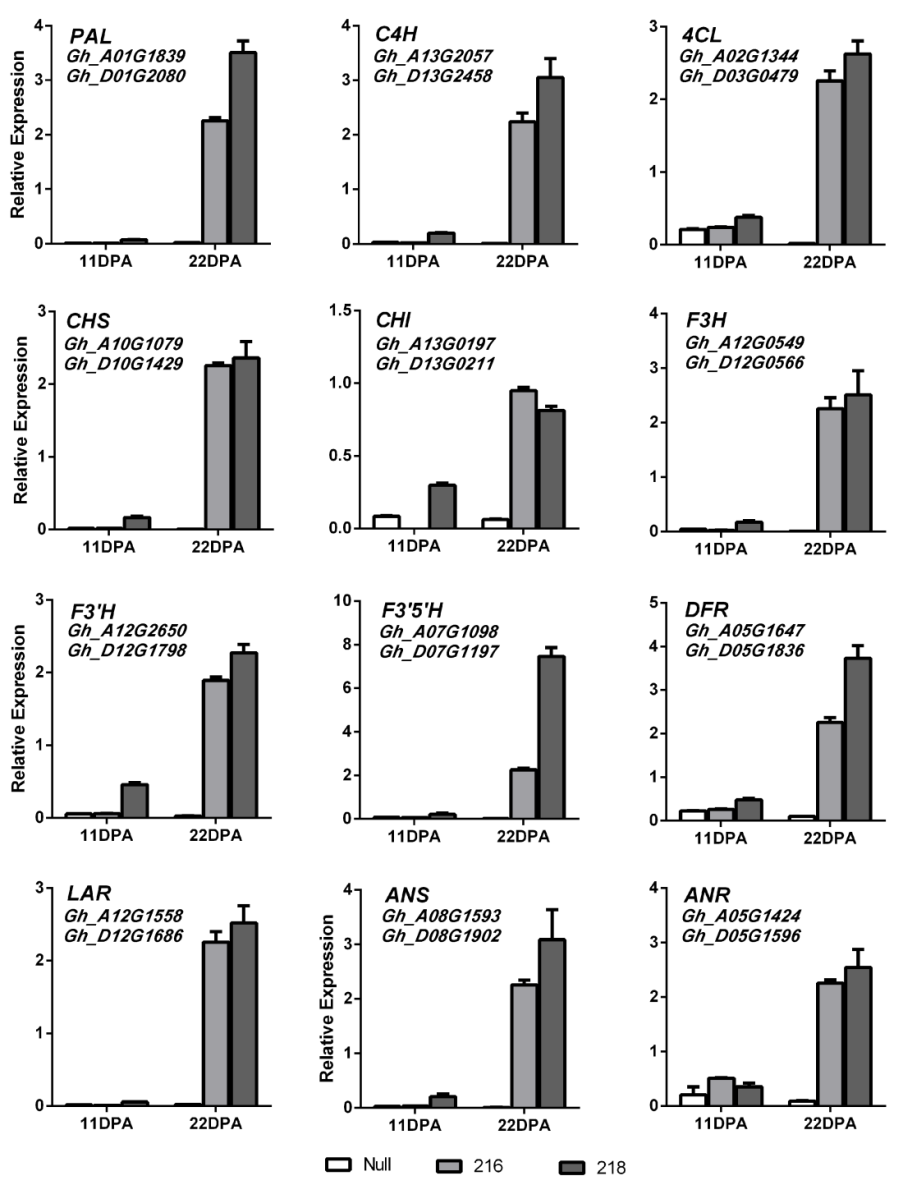


**Figure S8. qRT-PCR analysis of PA structural genes in *FbL2A:GhTT2-3A* transgenic fibers**

PA synthases are abbreviated as in Fig. 2. Primers are designed to amplify homeologous gene pairs from A and D sub-genome as indicated.


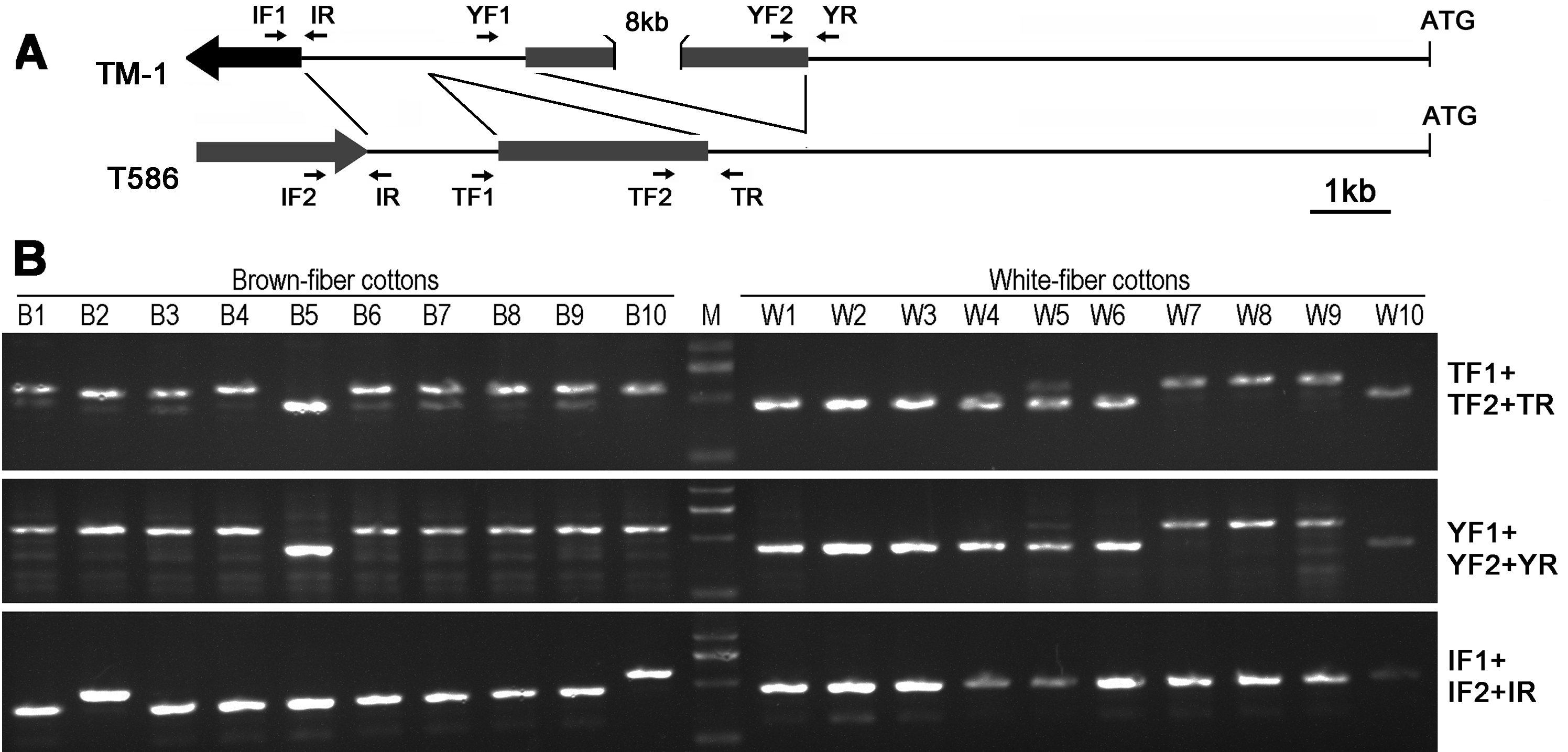


**Figure S9. Divergence of *GhTT2-3A* upstream sequence between brown- and white-fiber materials**

A, major sequence variations between T586 (brown fiber) and TM-1 (white fiber) upstream to the initiation ATG of *GhTT2-3A*. The two sequences are drawn in scale, and bars and arrows represent the differentially inserted retrotransposons and inverted fragments, respectively. Primers used to amplify differential sequences are marked on the corresponding positions. B, Amplification of the differential sequences in different brown- and white-fiber materials. The special retrotransposons in T586 and YM1, and the conjuction of genomic inversion are amplified using three primer combinations (TF1+TF2+TR, YF1+YF2+YR and IF1+IF2+IR, respectively). B1-B10 represent brown-fiber materials Zhongmiansuo No. 81, lines from National Medium-term Gene Bank of Cotton in China (Accession Nos. 140422, 140476, 140464, 140460, 140474, 140468, 140465 and 140469) and T586, respectively. W1-W10 represent white-fiber materials Yumian No.1, J14, Zhongmian No. 35 and No. 60, Ekanmian No. 8 and No. 24, Lumian No. 29, Alala maxa, Jingnong No. 7 and semi-wild race Latifolium TX-48 , respectively.
