# Supplementary material for: Up‐regulation of GhTT2‐3A in cotton fibres during secondary wall thickening results in brown fibres with improved quality
Source: Plant Biotechnol J. 2018 Apr 2;16(10):1735–47. doi: 10.1111/pbi.12910 (PMC6131414; doi:10.1111/pbi.12910)
Supplement: Supplementary file 2 — Table S1 Markers used for fine mapping of Lc1. Table S2 Protein‐coding genes annotated in Lc1 region. Table S4 Two‐way ANOVA in lint percentage and fiber quality traits of FbL2A:GhTT2‐3A fibers and controls from two harvest times. Table S5 Primers used in cloning. Table S6 Primers used in qRT‐PCR analysis. [file PBI-16-1735-s003.docx]

**Table S1. Markers used for fine mapping of *Lc1***

| Markers | Type | Primers (5’-3’) | Position in A2 genome  (CGP-BGI v2.0) | Position in AD1 genome  (JGI v1.1 ) |
| --- | --- | --- | --- | --- |
| Pec53L | SNP | CACCAAAATTCTAAAATCCAAACAC  CTACCACTCGAGCAATAACTTATT | Ca1: 1244863*–*1244776 | A07: 1088921*–*1088834 |
| TT2-1A | Indel | TTTGAGAAAGAGAGCCAAAGC  GCTTCCACTGTTGCAATATCA | Ca1: 2639448*–*2639732 | A07: 2230500*–*2230603 |
| INB | SNP | CCTCCATGACCCGACAAAAT  CCCAATTTACTTAACACTCC | Ca1: 2676361*–*2676462 | A07: 2275096*–*2275194 |
| TT2-3A | Indel | AAAGTATAATATAATGTCGGA  CAACATGGACAGTAACATGTGAA | Ca1: 2688234*–*2688329 | A07: 2297849*–*2297930 |
| MSIC10A | SNP | GAATTAACCGTTGATCGAAATTTC  TGATTAATTGATCTAGTTCAGTCG | Ca1: 2712542*–*2712498 | A07: 2321863*–*2321801 |
| BAC08 | SSR | TCTTTATACCTATCATTTCCCATG CATCAAAATCAGAAGAAAAACAGTC | Ca1: 2723372*–*2723496 | A07: 2333105*–*2333253 |
| PHDA | Indel | GTTGAAGTACCGGATTGTAAAAAG  CATTGGACCATTACTTTTCTGTTT | Ca1: 2749252*–*2749407 | A07: 2365814*–*2365955 |
| CIR238 | SSR | TCAACTCACCGATTACAC  TCTTCATTCGGGCTT | Ca1: 3139244*–*3139566 | scaffold_54: 76153*–*75831 |
| NAU3181 | SSR | CCCTTATGTCCACGATCTTC  GGAAGGAACAGGTGCTACAA | Ca1: 49604238*–*49604027 | A07: 3490288*–*3490502 |

**Table S2. Protein-coding genes annotated in *Lc1* region**

| Genes | At1 | Dt1 | A2 | D5 | Annotation |
| --- | --- | --- | --- | --- | --- |
| G1 | *Gohir.A07G020400* | *Gohir.D07G020100* | *Cotton_A_05639* | *Gorai.001G020900* | Mechanosensitive ion channel protein 10 |
| G2 | *-* | *-* | *-* | *Gorai.001G020800* | 40S ribosomal protein S3 |
| G3 | *Gohir.A07G020300* | *Gohir.D07G020000* | *Cotton_A_05640* | *Gorai.001G020700* | Peroxisome assembly protein 10 |
| G4 | *Gohir.A07G020200* | *Gohir.D07G019900* | *Cotton_A_05641* | *Gorai.001G020600* | Transcription factor TT2 |
| G5 | *Gohir.A07G020000* | *Gohir.D07G019800* | *GaTT2-2* | *Gorai.001G020500* | Transcription factor TT2 |
| G6 | *Gohir.A07G019900* | *Gohir.D07G019700* | *Cotton_A_05642* | *Gorai.001G020400* | Transcription factor TT2 |
| G7 | *Gohir.A07G020100Gohir.A05G287800 Gohir.A12G028500 Gohir.A10G201500 Gohir.A11G223000*  *Gohir.A04G061600*  *Gohir.A12G032500*  *Gohir.A08G051700 Gohir.A12G115900*  *Gohir.A13G119400*  *Gohir.A10G000100*  *Gohir.A01G000200* | *-*  *Gohir.D05G289000Gohir.D05G384500*  *Gohir.D01G152400*  *Gohir.D09G028700* | *-*  *Cotton_A_32686* | *-*  *Gorai.009G304100* | 50S ribosomal protein L9 |

Note: No ortholog of G7 was identified in A2, D5 and Dt1 (sub)genomes, while the paralogs are underlined.

**Table S4. Two-way ANOVA in lint percentage and fiber quality traits of *Fbl2A:GhTT2-3A* lines and controls**

| **Source** | **SS** | **DF** | **MS** | **F value** | **P value** | **Significance** |
| --- | --- | --- | --- | --- | --- | --- |
| **Lint percentage** | | | | | |  |
| **Genotype** | 337.4 | 3 | 112.5 | 38.87 | < 0.0001 | **** |
| **Harvest time** | 3.227 | 1 | 3.227 | 6.981 | 0.0296 | * |
| **Interaction** | 4.414 | 3 | 1.471 | 3.183 | 0.0846 | ns |
| **Block** | 23.14 | 8 | 2.893 | 6.259 | 0.0089 | ** |
| **Error** | 3.697 | 8 | 0.4622 |  |  |  |
| **Fiber length** | | | | | |  |
| **Genotype** | 244.6 | 3 | 81.52 | 103.4 | 0.0001 | **** |
| **Harvest time** | 1.293 | 1 | 1.293 | 6.925 | 0.0301 | * |
| **Interaction** | 6.218 | 3 | 2.073 | 11.10 | 0.0032 | ** |
| **Block** | 6.306 | 8 | 0.7883 | 4.223 | 0.0287 | * |
| **Error** | 1.493 | 8 | 0.1867 |  |  |  |
| **Fiber uniformity** | | | | | |  |
| **Genotype** | 180.2 | 3 | 60.08 | 100.5 | 0.0001 | **** |
| **Harvest time** | 10.98 | 1 | 10.98 | 23.48 | 0.0013 | ** |
| **Interaction** | 1.073 | 3 | 0.3576 | 0.7651 | 0.5448 | ns |
| **Block** | 4.784 | 8 | 0.5980 | 1.279 | 0.3680 | ns |
| **Error** | 3.740 | 8 | 0.4675 |  |  |  |
| **Fiber strength** | | | | | |  |
| **Genotype** | 188.4 | 3 | 62.81 | 90.90 | < 0.0001 | **** |
| **Harvest time** | 13.14 | 1 | 13.14 | 41.83 | 0.0002 | *** |
| **Interaction** | 15.05 | 3 | 5.016 | 15.97 | 0.0010 | *** |
| **Block** | 5.528 | 8 | 0.6910 | 2.199 | 0.1429 | ns |
| **Error** | 2.513 | 8 | 0.3142 |  |  |  |

**Table S5. Primers used for cloning**

| Primers | Sequences（5’-3’） | Usage |
| --- | --- | --- |
| GoTT2-1F  GoTT2-1R | ACAAAAGAGCGATATGG  CCACCCTATGTTTGGAAATTG | To amplify the coding regions of *GoTT2-1s* from cotton genomes |
| GoTT2-2F  GoTT2-2R | CTTGAGGTGTTAGTTTC  CTTATTTCACCATCATGG | To amplify the coding regions of *GoTT2-2s* from cotton genomes |
| GoTT2-3F  GoTT2-3R | ATGGGAAGAAGTCC  TCATGGCCATTCATCAG | To amplify the coding regions of *GoTT2-3s* from cotton genomes |
| GoTT2-4F  GoTT2-4R | ATGACGTCCACCCCCAATTATAATA  CCTAACAGTCAAATCTTATCAC | To amplify the coding regions of *GoTT2-4s* from cotton genomes |
| GoTT2-5F  GoTT2-5R | GTAAAAATATGGGGAGGAGG  CCTAACAGTCAAATCTTATCAC | To amplify the coding regions of *GoTT2-5s* from cotton genomes |
| GhTT2-1DF  GhTT2-1DR | ggatccACAAAAGAGCGATATGG  CCACCCTATGTTTGGAAATTG | To amplify cDNA sequence of *GhTT2-1D* |
| GhTT2-2DF  GhTT2-2DR | ggatccATGGGGAAGAGTTCATGTTG  TCATGGCCAGTCGTTAGTGT | To amplify cDNA sequence of *GhTT2-2D* |
| GhTT2-3AF  GhTT2-3AR | ggatccaatATGGGAAGAAGTCC  TCATGGCCATTCATCAG | To amplify cDNA sequence of *GhTT2-3A* |
| GhTT2-4DF  GhTT2-4DR | ggatccaatATGGGGAGGAGGCCTTG  GGATTACTAATTAGTTGATCCATTC | To amplify cDNA sequence of *GhTT2-4D* |
| GhTT2-5DF  GhTT2-5DR | ggatccATGGGAAGGAGTCCTTGTTG  GCTCATTCAATTCCATTTTCAAG | To amplify cDNA sequence of *GhTT2-5D* |
| Fbl A2-F  Fbl A2-R | aagcttGATGGCGTTCAGGAGCTTGG  ggatccAACCGAAATACAAAGCAAAG | To amplify the Fbl A2 promoter |
| SCFP-F  SCFP-R | aagcttACAACTTTTCTCTACCAATC  ggatccAAGCACAAGCTTTTTAGG | To amplify the SCFP promoter |
| ANR-F  ANR-R | aagcttTTTTTACTTTTTCTCGATTATC  ggatccGCTTCTGTCTTAATCTGTGT | To amplify the ANR promoter |
| LAR-F  LAR-R | aagcttCATCACACTCACGAGTTTC  ggatccGGTTAGCGTTATTGCAAG | To amplify the LAR promoter |
| GhbHLH130D-F  GhbHLH130D-R | ggatccATGGCTGTGCCGCCTAGTA  gaattcGTGGATTAGAATGAAGGTATC | To amplify cDNA sequence of *GhbHLH130D* |
| GUS-Up  GUS-Dn | ATGTTACGTCCTGTAGAAACC  AAGAGAGGTTAAAGCCGACAG | To amplify GUS probe for Southern blotting |

**Table S6. Primers used for real-time PCR**

| Target gene(s) | Primers | Sequences（5’-3’） |
| --- | --- | --- |
| *GhTT2-1A* | GhTT2-1A-U  GhTT2-1A-D | AGCTACCTGCAGCTCAACAA  TGCCTCATCAAAGGCTAAGG |
| *GhTT2-1D* | GhTT2-1D-U  GhTT2-1D-D | AGCTACCTGCAGCTCAACAT  TGCCTCATCAAAGGCTAAGG |
| *GhTT2-2A* | GhTT2-2A-U  GhTT2-2A-D | ACAGTGATGGCGGCTCCAA  GCCAAAGAGTCCCAACCTG |
| *GhTT2-2D* | GhTT2-2D-U  GhTT2-2D-D | ACAGTGATGGCGGCTCGGT  GCCAAAGAGTCCCAACCTG |
| *GhTT2-3A* | GhTT2-3A-U  GhTT2-3A-D | TACAGTGATGGCGGCTCTG  TGATTCCAAATCAGTGGCAC |
| *GhTT2-3D* | GhTT2-3D-U  GhTT2-3D-D | TACAGTGATGGCGGCTCAA  TGATTCCAAATCAGTGGCAC |
| *GhTT2-4A* | GhTT2-4A-U  GhTT2-4A-D | CCGAAACTCTAGTCAACA  CGTTTTGGGTTCGATAC |
| *GhTT2-4D* | GhTT2-4D-U  GhTT2-4D-D | CTCACAATGTGATTTGGACC  CATTATCCTCATTGATGGCTG |
| *GhTT2-5A* | GhTT2-5A-U  GhTT2-5A-D | CCAATGGTAATGGTAGTG  GGTGATCCCCATGATTTG |
| *GhTT2-5D* | GhTT2-5D-U  GhTT2-5D-D | GCTAATGGTAATGGTAGTG  GCTAATGGTAATGGTAGTG |
| *GhACT4* | GhACT4-U  GhACT4-D | TTGCAGACCGTATGAGCAAG  ATCCTCCGATCCAGACACTG |
| *GhUBQ14* | GhUBQ14-U  GhUBQ14-D | CAACGCTCCATCTTGTCCTT  TGATCGTCTTTCCCGTAAGC |
| *Gh_A01G1839*  *Gh_D01G2080* | PAL-U  PAL-D | AGCTTGGAACTGGGTTGTTG  AGCACCATTCCAACCCTTTA |
| *Gh_A13G2057*  *Gh_D13G2458* | C4H-U  C4H-D | TTTGGGTCGTTTGGTACAGA  AAAATTGCCTTGGCTTAGCA |
| *Gh_A02G1344*  *Gh_D03G0479* | 4CL-U  4CL-D | AAGGTGCACTTTGTTCATGC  cgttgcaatttaaaagccaaat |
| *Gh_A10G1079*  *Gh_D10G1429* | CHS-U  CHS-D | CAGGAGAAGGACTGGAGTGG  AGCAGCAACACTATGGAGCA |
| *Gh_A13G0197*  *Gh_D13G0211* | CHI-U  CHI-D | ATGGAGTTTCTCCTCCAGCA  GGTTTTTCACTGTCGACTCCA |
| *Gh_A12G0549*  *Gh_D12G0566* | F3H-U  F3H-D | CTGAAGAAGCTGGCCAAAGA  TGCAAGGATTTCCTCCAATG |
| *Gh_A12G2650*  *Gh_D12G1798* | F3'H-U  F3'H-D | GCTGATGTTAGGGGCAATGA  ctcaccatgaaacgacaacg |
| *Gh_A07G1098*  *Gh_D07G1197* | F3'5'H-U  F3'5'H-D | AAACATGGATGAGGCCTTTG  gcaagggatgtgcTTAGGAA |
| *Gh_A05G1647*  *Gh_D05G1836* | DFR-U  DFR-D | CATGTTCGTAGGAGCTGTCG  ggtaggcactcaattgttgaaa |
| *Gh_A12G1558*  *Gh_D12G1686* | LAR-U  LAR-D | GAATGAGCCATTCCGAACAT  GCTTCGACTACTGGCTTTGG |
| *Gh_A08G1593*  *Gh_D08G1902* | ANS-U  ANS-D | GCCACCGAAGGATAAGATCA  TGGGTCTTCCTGAACAGCTT |
| *Gh_A05G1424*  *Gh_D05G1596* | ANR-U  ANR-D | TGGGATCGAGGAAATCTACG  accataatcattggggaagc |
